# The Demands of Professional Rugby League Match-Play: a Meta-analysis

**DOI:** 10.1186/s40798-019-0197-9

**Published:** 2019-06-11

**Authors:** Daniel J. Glassbrook, Tim L. A. Doyle, Jacqueline A. Alderson, Joel T. Fuller

**Affiliations:** 1 Department of Health Professions, Faculty of Medicine and Health Sciences, Macquarie University, Macquarie Park, Australia; 2 School of Human Sciences, The University of Western Australia, Perth, Australia

**Keywords:** Football, Playing position, Global positioning system, Performance, Collisions

## Abstract

**Background:**

Rugby league is a collision sport, where players are expected to be physically competent in a range of areas, including aerobic fitness, strength, speed and power. Several studies have attempted to characterise the physical demands of rugby league match-play, but these studies often have relatively small sample sizes based on one or two clubs, which makes generalisation of the findings difficult. Therefore, the aim of this review was to synthesise studies that investigated the physical demands of professional rugby league match-play.

**Methods:**

SPORTDiscus, CINAHL, MEDLINE (EBSCO) and Embase (EBSCO) databases were systematically searched from inception until October 2018. Articles were included if they (1) recruited professional rugby league athletes aged ≥ 18 years and (2) provided at least one match-play relevant variable (including playing time, total and relative distance, repeat high-intensity efforts (RHIE), efforts per RHIE, accelerations and decelerations, total and relative collisions). Meta-analyses were used to provide pooled estimates ± 95% confidence intervals.

**Results:**

A total of 30 studies were included. Pooled estimates indicated that, compared to adjustables and backs, forwards have less playing time (− 17.2 ± 5.6 and − 25.6 ± 5.8 min, respectively), cover less ‘slow-speed’ (− 2230 ± 735 and − 1348 ± 655 m, respectively) and ‘high-speed’ distance (− 139 ± 108 and − 229 ± 101 m, respectively), but complete more relative RHIEs (+ 0.05 ± 0.05 and + 0.08 ± 0.04 per minute, respectively), and total (+ 12.0 ± 8.1 and + 12.8 ± 7.2 collisions, respectively) and relative collisions (+ 0.32 ± 0.22 and + 0.41 ± 0.22 collisions per minute, respectively). Notably, when the distance was expressed relative to playing time, forwards were not different from adjustables and backs in slow-speed (*P* ≥ 0.295) and high-speed (*P* ≥ 0.889) relative distance. The adjustables and backs subgroups were similar in most variables, except playing time (shorter for adjustables, − 8.5 ± 6.2 min), slow-speed distance (greater for adjustables, + 882 ± 763 m) and total relative distance (greater for adjustables, + 11.3 ± 5.2 m·min^−1^). There were no significant differences between positional groups for efforts per RHIE, accelerations and decelerations (*P* ≥ 0.745).

**Conclusions:**

These results indicate the unique physical demands of each playing position and should be considered by strength and conditioning and tactical coaches when planning for professional rugby league performance.

**Protocol Registration:**

https://osf.io/83tq2/

**Electronic supplementary material:**

The online version of this article (10.1186/s40798-019-0197-9) contains supplementary material, which is available to authorized users.

## Key Points

- Forwards spend the least amount of time on the field and cover the least ‘slow-speed’ and ‘high-speed’ total distance but cover the same relative distance per minute.
- Forwards complete the most repeat high-intensity efforts per minute of playing time and experience the most total and relative collisions.
- Adjustables and backs subgroups were similar in most variables, except playing time (shorter for adjustables), slow-speed distance (greater for adjustables) and total relative distance (greater for adjustables).
- There were no significant differences between any positional group for total efforts per repeat high-intensity effort, total accelerations and total decelerations.

## Background

Rugby league is a team sport involving regular and purposeful bouts of high-intensity activity (e.g. accelerations and sprinting), periods of low-intensity activity (e.g. walking and jogging) and collisions of great force [1, 2]. Two teams of 13 players (and 5 interchanges) compete to carry the ball and place it behind the opposition team’s goal line. Rugby league is played at amateur [3], semi-professional [4] and professional [5] levels, and predominantly in Australia and England, with the Australian National Rugby League and English Super League being the two premier competitions in the world. In professional rugby league, games comprise of two 40-min halves, separated by a 10-min half-time interval, and players are expected to be physically competent in a range of areas, including aerobic fitness, speed, agility, strength and power [6, 7]. Understanding the demands of professional rugby league match-play is important for both sport scientists and coaches and facilitates improved training design for athletes playing at the highest level.

Characterising the demands of professional rugby league match-play has been attempted by several authors [8–20]; however, these studies often have relatively small sample sizes based on players from only one or two clubs (mean ± standard deviation: 24 ± 13 players). Additionally, a number of reviews have attempted to synthesise the findings of these small or limited-scale studies to determine a more precise and generalisable characterisation of rugby league match-play [6, 21–28]. However, these reviews have included studies based on non-professional competitions that involve a lower match standard than professional rugby league. Moreover, of the reviews completed in this area, there has only been one meta-analysis completed [28]. In 2016, Hausler et al. [28] completed a quantitative synthesis of four variables (total distance, relative distance, number of repeat high intensity (RHIE) bouts and number of efforts per RHIE bout) across three positional groups (forwards, backs and adjustables) and three levels of play (elite, sub-elite and junior). Significant differences were evident for all four variables, but several additional variables (e.g. high- and low-speed distance and collisions) were not considered, and meta-analysis was not performed for positional group differences in the number of RHIE bouts, and number of efforts per RHIE bout. These variables provide additional important information about rugby league match demands that were not captured by the previous review. For example, high-speed running demands influence the extent of muscle damage experienced by players during matches [15] and collisions impact on skill efficiency and the ability of players to maintain high running intensities [29–31]. Improving our understanding of how they may differ across playing positions should further improve training design. Therefore, the aim of this review was to systematically characterise the demands of only professional rugby league match-play, by expanding on previous research and further synthesising the literature across a greater range of variables than has been previously conducted.

## Methods

### Systematic Review Protocol

The protocol for this review was prospectively registered with the Open Science Framework (DOI: 10.17605/OSF.IO/83TQ2), and the review was performed in accordance with PRISMA guidelines [32].

### Eligibility Criteria

Articles were eligible for inclusion in this review if they (1) included professional level rugby league athletes aged ≥ 18 years and (2) provided at least one match-play relevant variable (including playing time, total distance, relative distance, total RHIE, total efforts per RHIE, total accelerations, total decelerations, total collisions or total relative collisions). Articles were excluded if they were (1) published in a non-English language or (2) only available in conference abstract or conference proceedings format.

### Search Strategy

SPORTDiscus, CINAHL, MEDLINE (EBSCO) and Embase (EBSCO) electronic databases were systematically searched from inception up to 29 October 2018. The strategy was formed by adopting the following search string:

(rugby or football) AND (global position* system* OR gps inertial* or accelerometer* OR telemetry OR geographic information system* OR remote sens* OR time stud* OR video stud* OR time AND motion stud*) AND (((speed OR distance OR acceleration* OR sprint* OR run* OR movement OR activity)) OR (heart rate* OR pulse rate* OR respiratory rate*) OR (physiolog* OR metabol*) OR mechanic*)

### Study Selection

Search results were exported to a reference manager library (Endnote, X8) and then uploaded to the Covidence web-based systematic review tool (available at www.covidence.org). Duplicate records were removed before the title and abstract of the remaining records were screened for eligibility independently by two authors (DJG and JTF). Full-texts of potentially eligible articles were retrieved before a final eligibility assessment was completed by one author (DJG) and checked by another (JTF). Any discrepancies between reviewer eligibility assessments were resolved through a discussion amongst all authors until a consensus was reached. A comprehensive manual search of the reference list of all retrieved papers was also performed to identify any additional relevant articles.

### Data Extraction

Data relating to participant characteristics (age, height, body mass and professional league), global positioning system (GPS) or camera specifications (brand, model and sampling frequency), playing time (min) and match-play relevant variables (playing time, distances, accelerations, decelerations, RHIE and collisions) were extracted. The distance was considered the absolute (m) or relative (m·min^−1^) distance covered in total or within defined GPS speed zones. All speed zones were converted to km·h^−1^ and categorised as slow speed (0–18 km·h^−1^) or high speed (> 18 km·h^−1^) to facilitate comparison across studies. Previous research indicates that 18 km·h^−1^ is a common threshold for high-speed running [8, 10, 13–15, 18, 33–39]. The number of accelerations and decelerations were defined based on GPS thresholds of > 2.78 m·s^−2^ and < − 2.78 m·s^−2^, respectively [11]. Both the total number of RHIEs and the total number of efforts within each RHIE were considered. A RHIE was defined as three or more high-intensity efforts, such as sprints, tackles, or collisions with less than 21 s recovery between efforts [40], and this was consistent across all studies included in this analysis. Collisions were considered the absolute or relative (n·min^−1^) number of collisions in total or within low (0–8 g) and high (> 8 g) impact zones. These collisions zones were based on a previous study [9] and only included two zones because discrepancies in zone definitions within the literature prevented consideration of additional or intermediate zones. Collisions included efforts such as player hit-ups or tackles but did not include accelerations, decelerations, or change of directions while running. Where collisions were presented only in multiple magnitude zones, data were combined to determine the total collisions.

Whenever possible, data were extracted by playing position. Playing positions were grouped into ‘forwards’ (prop, second row, lock), ‘adjustables’ (halfback, hooker, five-eighth, fullback) and ‘backs’ (centre, wing). ‘All positions’ data were considered because playing position-specific data were not always available. Only data pertaining to a full match were considered (inclusive of substitute players and player interchanges). However, when the results were presented as data for the first half and second half, these data were combined to represent the full match. If results for interchange players were presented separately from players who played a complete match, then both results were included in the meta-analysis.

### Assessment of Methodological Quality

In line with similar reviews [22, 28], a modified assessment scale of downs and black [41], was used to evaluate the methodological quality of each included study. Of the 27 criteria, the 12 that were relevant to the study designs included in this review were used. Quality of reporting (1–4, 6, 7, 10), external validity (11, 12) and internal validity bias (16, 18, 20) were assessed for each included study.

### Statistics

Meta-analyses were performed using random-effects models with the Metafor statistical package in R software (version 3.4.3, R Foundation for Statistical Computing). Separate meta-analyses were performed for each individual outcome measure and individual study effects were weighted using the inverse variance method. Variances were calculated from the standard deviation, standard error, or confidence interval, and the number of observations reported by each study. We assumed only one observation per player for studies that only reported the number of players without including the total number of observations. We used the largest standard deviation across studies to calculate a conservative estimate of the variance for studies that did not report a standard deviation, standard error or confidence interval. Sub-group analyses were performed based on playing position. Multiple study effects and variances from the same study were included in the meta-analysis provided that the effects and variables resulted from independent observations (i.e. National Rugby League and Super League matches [18, 42], or trial and competition matches [43]). All data are presented as mean, or mean ± standard deviation, or mean ± 95% confidence interval (95%CI).

Statistical heterogeneity within each meta-analysis was investigated using Cochran’s *Q* and *I*^2^ statistics. Statistical heterogeneity was considered low (*I*^2^ < 25%), moderate (*I*^2^ = 25–49%) and high (*I*^2^ > 50%) [44]. Publication bias was not assessed because there was no reason to expect that studies finding lower or higher estimates of the physical demands during rugby league match-play would be more or less likely to be published.

## Results

### Identification and Selection of Studies

**Fig. 1:**
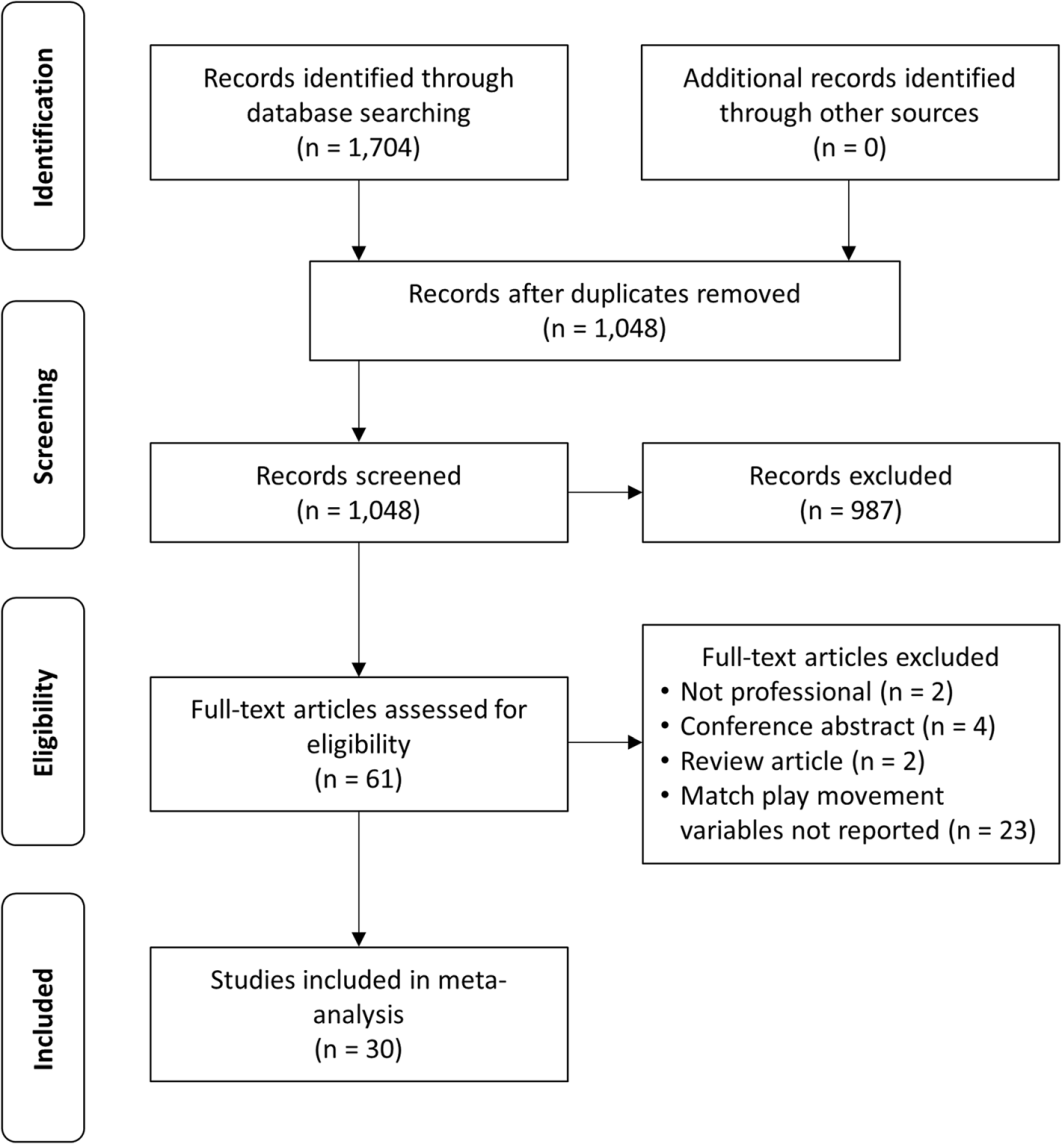
Results of study selection process

The original database search identified 1704 records. A total of 30 studies met the eligibility criteria and were included in the review [8–20, 33–40, 42, 43, 45–51]. An overview of the study selection process is presented in Fig. 1.

### Study Characteristics

A summary of the characteristics of all included studies is presented in Table 1. A total of 686 professional players were included across the 30 studies (21 ± 14 per study). A total of 230 forwards (12 ± 8), 60 adjustables (5 ± 2) and 120 backs (7 ± 4) were included. The remaining 276 (31 ± 14) players were not described by position and could only be included in an all positions subgroup. A total of 4246 observation files were included across the 30 studies (129 ± 143 per study). The total observation files for each position group were 1775 forwards (71 ± 66), 624 adjustables (42 ± 34), 1063 backs (46 ± 35) and 784 (196 ± 300) all positions. Three papers [8, 18, 33] did not report how many participants were included in the study, and instead provided only the number of observation files used in analysis. Additionally, four papers [14, 42, 45, 46] only reported the number of participants, and not how many observation files were used in analysis.

**Table 1:** Characteristics of included studies

Study	Participants (*n*)	Observations/files (*n*)	Competition (year)	Device details	Variables extracted
Austin et al. (2011) [40]	F: 5 AD: 5 B: 5 Total: 15	F: 25 AD: 25 B: 25 Total: 75	NRL (2008)	Cameras: DZGX060SW, Hitachi	Total RHIE
Austin and Kelly (2013) [33]	NR	F: 103 B: 82 Total: 185	NRL (NR)	GPS: SPI Elite, 5 Hz, GPSports	Playing time, total distance, total slow-speed distance, total high-speed distance, total relative distance
Austin and Kelly (2014) [8]	NR	F: 60 B: 75 Total: 135	NRL (NR)	GPS: SPI Elite, 5 Hz, GPSports	Playing time, total distance, total slow-speed distance, total high-speed distance, total relative distance
Cummins et al. (2016) [34]	F: 10 AD: 4 B: 4 Total: 18	F: 140 AD: 74 B: 59 Total: 273	NRL (2013)	GPS: SPI-ProX, 15 Hz, GPSports	Playing time, total distance, total relative distance
Cummins et al. (2017) [51]	F: 10 AD: 4 B: 4 Total: 18	F: 35 AD: 37 B: 58 Total: 130	NRL (2013)	GPS: SPI-ProX, 15 Hz, GPSports	Playing time, total distance, total relative distance
Cummins et al. (2017) [51]	F: 10 AD: 4 Total: 14	F: 105 AD: 37 Total: 142	NRL (2013)	GPS: SPI-ProX, 15 Hz, GPSports	Playing time, total distance, total relative distance
Cummins and Orr (2015) [9]	F: 13 AD: 5 B: 5 Total: 23	F: 184 AD: 97 B: 78 Total: 359	NRL (NR)	GPS: SPI-Pro X II, 15 Hz, GPSports Accelerometer: SPI-Pro X II, 100 Hz, GPSports	Playing time, total collisions, total ‘low impact’ collisions, total ‘high impact’ collisions, total relative collisions
Dempsey et al. (2018) [50]	F: 37 B: 20 Total: 57	F: 222 B: 120 Total: 342	International (2011 and 2012)	GPS: SPI Pro-X, GPSports	Playing time, total distance, total relative distance
Gabbett (2012) [35]	All: 37 Total: 37	F: 46 AD: 29 B: 29 Total: 104	NRL (NR)	GPS: MinimaxX, 5HZ, Catapult Accelerometer and Gyroscope, MinimaxX, 100 Hz, Catapult	Total RHIE
Gabbett (2013) [45]	All: 22 Total: 22	NR	NRL (NR)	GPS: Minimax Team 2.5, 5 Hz, Catapult	Playing time, total distance, total slow-speed distance, total high-speed distance, total relative distance, total slow-speed relative distance, total high-speed relative distance, total RHIE, total accelerations, total collisions, total ‘low impact’ collisions, total ‘high impact’ collisions, total relative collisions
Gabbett (2013) [43]	All: 24 Total: 24	F: 42 AD: 15 B: 19 Total: 76	NRL (NR)	GPS: Minimax Team 2.5, 5 Hz, Catapult Accelerometer, Gyroscope, and Magnetometer: Minimax Team 2.5, 100 Hz, Catapult	Playing time, total distance, total slow-speed distance, total high-speed distance, total relative distance, total slow-speed relative distance, total high-speed relative distance, total RHIE, total efforts per RHIE, total collisions, total ‘low impact’ collisions, total ‘high impact’ collisions, total relative collisions
Gabbett (2013) [43]	F: 7 AD: 5 B: 4 Total: 16	All: 61 Total: 61	NRL (NR)	GPS: Minimax Team 2.5, 5 Hz, Catapult	Playing time, total distance, total slow-speed distance, total high-speed distance, total relative distance, total slow-speed relative distance, total high-speed relative distance, total RHIE, total efforts per RHIE, total collisions, total ‘low impact’ collisions, total ‘high impact’ collisions, total relative collisions
Gabbett et al. (2011) [46]	All: 51 Total: 51	NR	NRL (2008–2010)	Camera: NR	Total collisions
Gabbett et al. (2012) [10]	All: 21 Total: 21	F: 46 AD: 29 B: 29 Total: 104	NRL (NR)	GPS: Minimax Team 2.5, 5 Hz, Catapult Accelerometer and Gyroscope, MinimaxX, 100 Hz, Catapult	Playing time, total distance, total slow-speed distance, total high-speed distance, total relative distance, total RHIE, total efforts per RHIE, total collisions, total ‘low impact’ collisions, total ‘high impact’ collisions, total relative collisions
Gabbett et al. (2014) [36]	F: 22 Total: 22	F: 226 Total: 226	NRL (NR)	GPS: Minimax Team 2.5, 10 Hz, Catapult Accelerometer and Gyroscope, MinimaxX, 100 Hz, Catapult	Total relative distance, total slow-speed relative distance, total high-speed relative distance
Kempton et al. (2017) [11]	ST: 29 LST: 25 All: 54 Total: 54	ST: 380 LST: 265 All: 645 Total: 645	NRL (2014)	GPS: SPI-Pro X, 15 Hz, GPSports	Playing time, total distance, total slow-speed distance, total high-speed distance, total relative distance, total slow-speed relative distance, total high-speed relative distance, total accelerations, total decelerations, total collisions, total relative collisions
Kempton et al. (2015) [47]	All: 25 Total: 25	F: 145 AD: 118 B: 121 Total: 384	NRL (2010–2011)	GPS: SPI-Pro, 5 Hz, GPSports	Playing time, total distance, total high-speed distance, total relative distance, total accelerations, total decelerations
King et al. (2009) [12]	F: 3 AD: 3 B: 3 Total: 9	F: 3 AD: 3 B: 3 Total: 9	NRL (2005)	Camera: Panasonic	Total distance
McLellan and Lovell (2012) [48]	F: 8 B: 7 Total: 15	F: 64 B: 56 Total: 120	NRL (NR)	GPS: SPI-Pro, 5 Hz, GPSports	Total distance, total collisions
McLellan and Lovell (2013) [37]	F: 6 B: 6 Total: 12	F: 30 B: 30 Total: 60	NRL (NR)	GPS: SPI-Pro, 5 Hz, GPSports	Total distance, total relative distance
McLellan et al. (2010) [38]	F: 8 B: 7 Total: 15	F: 8 B: 7 Total: 15	NRL (NR)	GPS: SPI-Pro, 5 Hz, GPSports	Total distance, total slow-speed distance, total high-speed distance
McLellan et al. (2011) [13]	F: 8 B: 7 Total: 15	F: 40 B: 35 Total: 75	NRL (NR)	GPS: SPI-Pro, 5 Hz, GPSports	Total distance, total slow-speed distance, total high-speed distance
McLellan et al. (2011) [39]	F: 8 B: 7 Total: 15	F: 8 B: 7 Total: 15	NRL (NR)	GPS: SPI-Pro, 5 Hz, GPSports	Total collisions
Murray et al. (2014) [14]	All: 31 Total: 31	NR	NRL (NR)	GPS: Minimax Team 2.5, 5 Hz, Catapult Accelerometer and Gyroscope, MinimaxX, 100 Hz, Catapult	Playing time, total distance, total slow-speed distance, total high-speed distance, total slow-speed relative distance, total high-speed relative distance, total RHIE, total collisions, total relative collisions
Oxendale et al. (2016) [15]	All: 17 Total: 17	F: 17 B: 11 Total: 28	NRL (NR)	GPS: Minimax Team 2.5, 10 Hz, Catapult	Playing time, total distance, total slow-speed distance, total high-speed distance, total relative distance, total slow-speed relative distance, total high-speed relative distance, total RHIE, total accelerations, total decelerations
Scott et al. (2018) [16]	F: 8 AD: 6 B: 8 Total: 22	F: 101 AD: 77 B: 102 Total: 280	NRL (2013)	GPS: SPI HPU, 15 Hz, GPSports	Playing time, total high-speed distance
Sirotic et al. (2009) [49]	All: 17 Total: 17	All: 39 Total: 39	NRL (2004–2005)	Camera: NR	Total relative distance
Sirotic et al. (2011) [17]	F: 16 AD: 8 B:15 Total: 39	All: 39 Total: 39	NRL (NR)	Camera: NV-DX100, Panasonic and GR-DVL 9800, JVC	Playing time, total relative distance
Twist et al. (2017) [42]	F: 8 AD: 3 B: 4 Total: 15	NR	Super League (NR)	GPS: Viper Pod 2, STATSports	Total distance, total relative distance, total slow-speed relative distance, total high-speed relative distance
Twist et al. (2014) [18]	NR	F: 29 AD: 29 B: 30 Total: 88	NRL (2011)	GPS: SPI-Pro, 5 Hz, GPSports Accelerometer, SPI-Pro, 100 Hz, GPSports	Playing time, total distance, total relative distance
Twist et al. (2014) [18]	NR	F: 39 AD: 23 B: 42 Total: 104	Super League (2011)	GPS: SPI-Pro, 5 Hz, GPSports Accelerometer, SPI-Pro, 100 Hz, GPSports	Playing time, total distance, total relative distance
Varley et al. (2014) [19]	F: 17 AD: 10 B: 9 Total: 36	F: 44 AD: 22 B: 28 Total: 94	NRL (NR)	GPS: Minimax Team 2.5, 5 Hz, Catapult	Playing time, total distance, total slow-speed distance, total high-speed distance, total relative distance, total slow-speed relative distance, total high-speed relative distance, total RHIE, total collisions, total low-impact collisions, total high-impact collisions, total relative collisions
Waldron et al. (2011) [20]	F: 4 AD: 3 B: 5 Total: 12	F: 13 AD: 9 B: 17 Total: 39	Super League (2010)	GPS: SPI-Pro, 5 Hz, GPSports Accelerometer, SPI-Pro, 100 Hz, GPSports	Playing time, total distance, total slow-speed distance, total high-speed distance, total relative distance

*NR*, not reported; GPS, global positioning system; *F*, forwards; *AD*, adjustables; *B*, backs; *All*, all positions; *ST*, successful team; *LST*, less-successful team; *RHIE*, repeat high intensity effort; *NRL*, National Rugby League

Twenty-seven of the 30 included studies reported on players from the Australian National Rugby League [8–19, 33–40, 43, 45–49, 51] and three studies reported on players from the English Super League competition [18, 20, 42]. One study also reported on international players in England during the Four Nations competition and Summer Tests [50]. Of the 30 studies, 5 studies [12, 17, 40, 46, 49] recorded movement variables from video analysis. The remaining studies utilized microtechnology (GPS and accelerometers, gyroscope and magnetometers) to measure movement variables.

### Methodological Quality

The methodological quality of studies was moderate to good, with scores ranging from 8 to 10 out of 12 (mean score 9) (Additional file 1: Table S1). All studies used convenience sampling from 1 to 2 clubs or representative teams, or did not report this clearly, which makes it difficult to generalise the results of any one study to the wider rugby league population. Eight studies did not provide a complete description of subject characteristics [12, 14, 18, 19, 37, 43, 45, 49]. Ten studies reported the actual probability values [9, 12, 17, 18, 20, 34, 48–51], whereas 12 studies reported effect sizes [10, 11, 14, 16, 19, 35, 36, 42, 43, 45–47].

### Meta-analysis Results

The following results are presented for each of the three position groups analysed, forwards, adjustables and backs. ‘All positions’ data can be found in the accompanying figures or additional files that are associated with each variable. The datasets used for analysis are available in Additional file 2: Appendix 1.

#### Playing Time

**Fig. 2:**
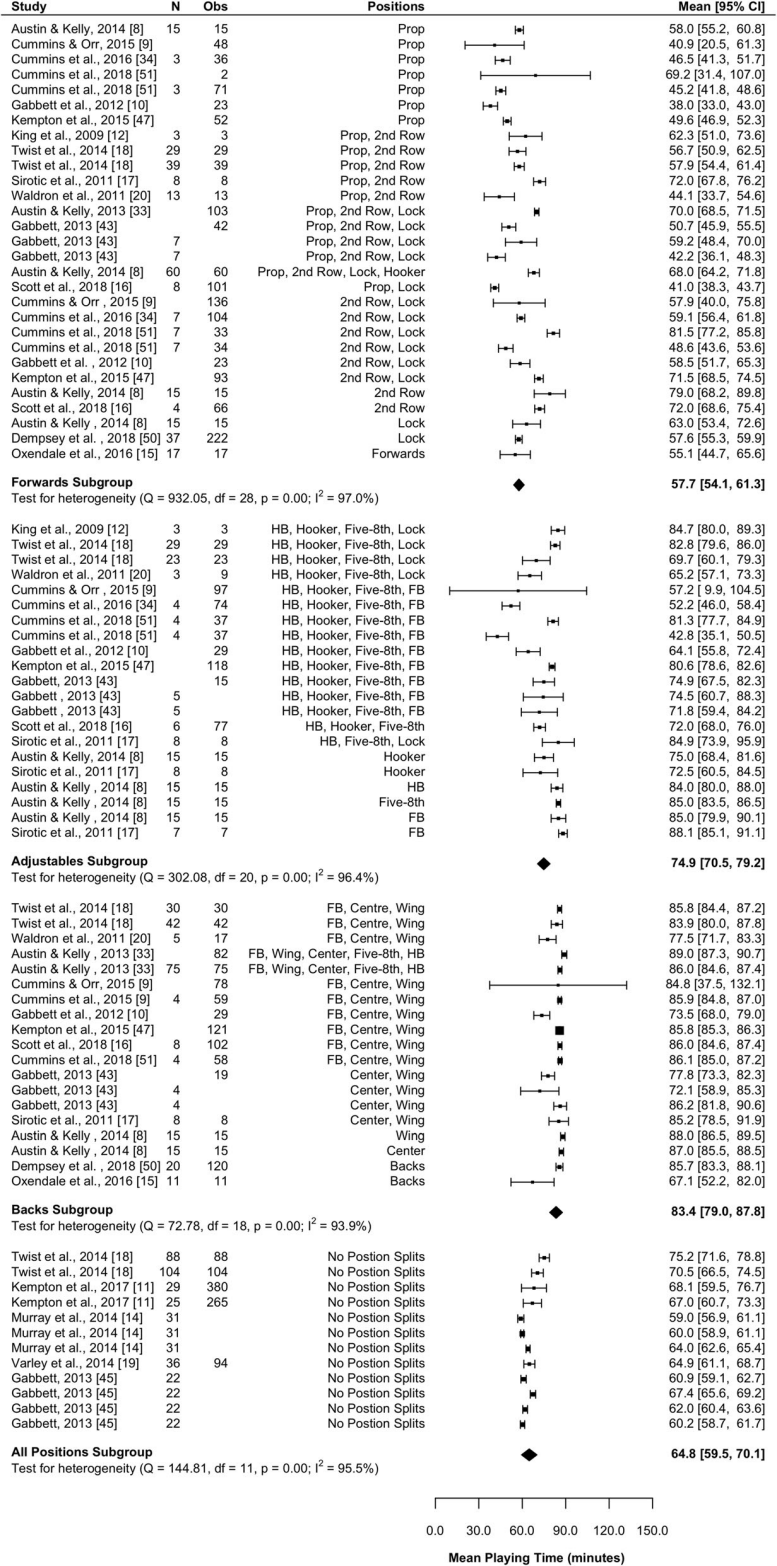
Meta-analysis of playing time. A forest plot (mean and 95% confidence intervals) was used to present the results of the meta-analysis and combined pooled estimates (random effects model). 2nd row, second row; HB, halfback; five-8th, Five-eighth; FB, fullback, Obs, observations; CI, confidence interval

Eighteen studies (60%) reported on playing time [8–11, 14–20, 33, 34, 43, 45, 47, 50, 51]. Results of the meta-analysis are presented in Fig. 2 and indicate that forwards spend significantly less (*P* < 0.001) time on the field during a match than adjustables (mean difference [95%CI] 17.2 [11.5–22.8] min) and backs (mean difference [95%CI] 25.6 [19.9–31.4] min). The adjustables also spent significantly less time on the field during a match than the backs (mean difference [95%CI] 8.5 [2.3–14.7] min; *P* = 0.007). All subgroups were associated with high heterogeneity (*I*^2^ > 93%).

#### Total Distance

**Fig. 3:**
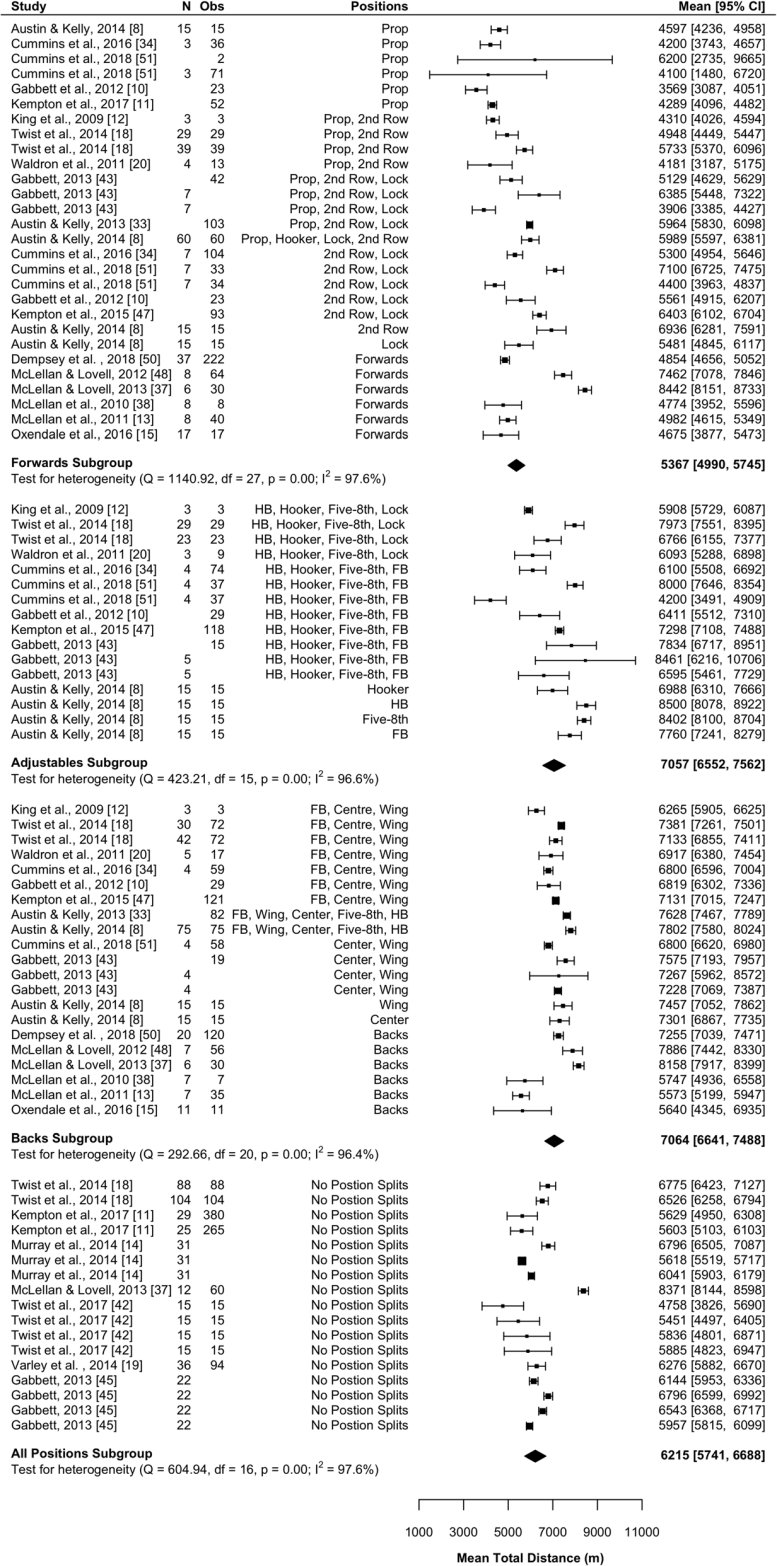
Meta-analysis of total distance. A forest plot (mean and 95% confidence intervals) was used to present the results of the meta-analysis and combined pooled estimates (random effects model). 2nd row, second row; HB, halfback; five-8th, five-eighth; FB, fullback, Obs, observations; CI, confidence interval

Total distance was the most reported variable with 21 of the 30 (70%) studies reporting this variable [8, 10–15, 18–20, 33, 34, 37, 38, 42, 43, 45, 47, 48, 50, 51]. Results of the meta-analysis are presented in Fig. 3 and indicate that forwards covered significantly less (*P* < 0.001) distance than adjustables (mean difference [95%CI] 1690 [1059–2320] m) and backs (mean difference [95%CI] 1697 [1129–2264] m). No significant difference was observed in total distance between adjustables and backs (*P* = 0.983). All subgroups were associated with high heterogeneity (*I*^2^ > 96%).

#### Total ‘Slow-Speed’ Distance

**Fig. 4:**
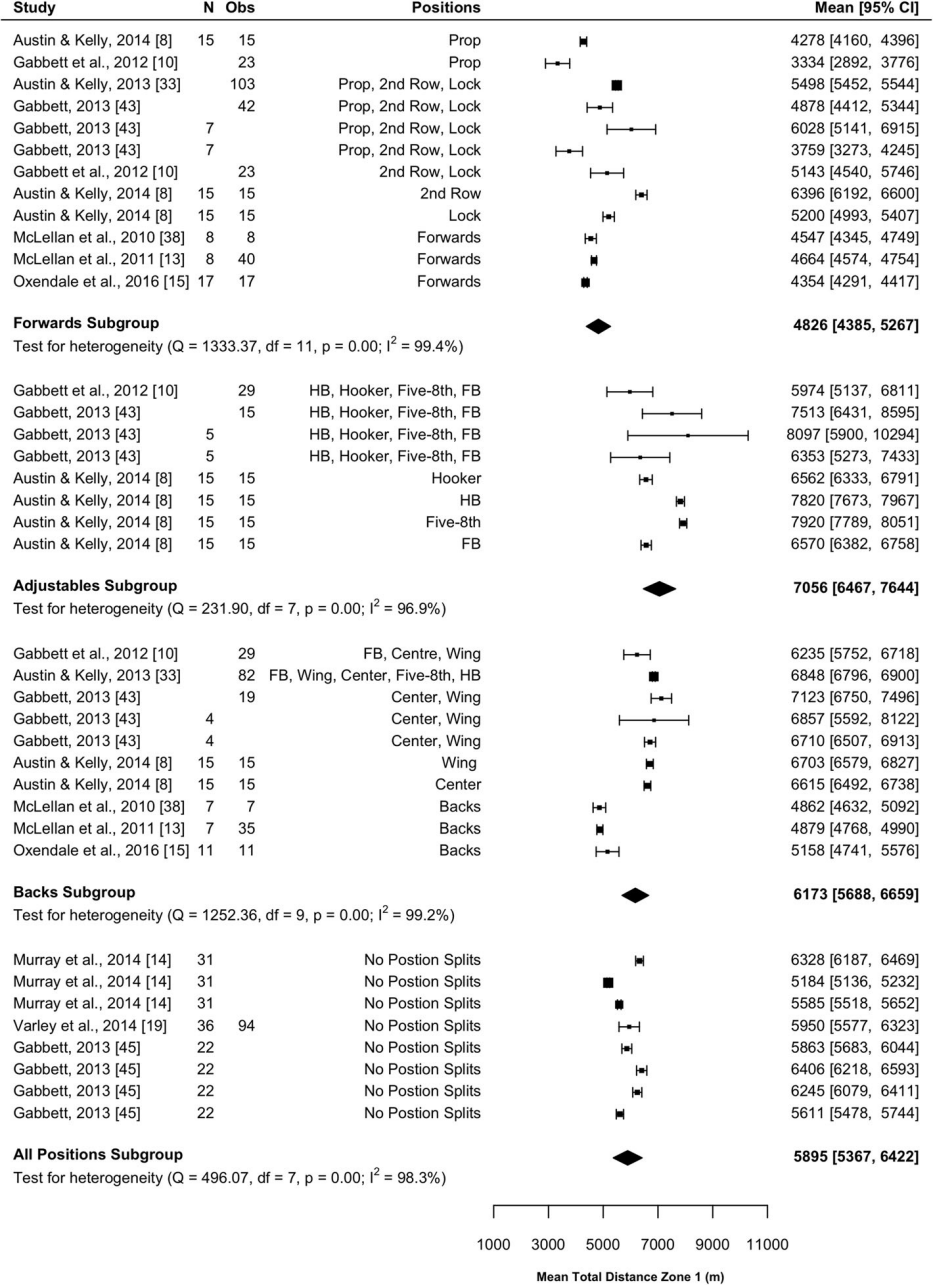
Meta-analysis of total slow-speed distance. A forest plot (mean and 95% confidence intervals) was used to present the results of the meta-analysis and combined pooled estimates (random effects model). 2nd row, second row; HB, halfback; five-8th, five-eighth; FB, fullback, Obs, observations; CI, confidence interval

Twelve studies (40%) reported on slow-speed distance [8, 10, 11, 13–15, 19, 20, 33, 38, 43, 45]. Nine studies reported distance at 0–18 km·h^−1^ [8, 10, 13–15, 33, 38, 43, 45]. Up to four speed zones were presented within slow speed from 0 to 18 km·h^−1^. Results of the meta-analysis are included in Fig. 4 and indicated that the forwards covered significantly less slow-speed distance (*P* < 0.001) than the adjustables (mean difference [95%CI] 2230 [1494–2965] m) and backs (mean difference [95%CI] 1348 [692–2003] m) subgroups. Adjustables covered significantly more slow-speed distance than backs (mean difference [95%CI] 882 [119–1645] m; *P* = 0.023). All subgroups were associated with high heterogeneity (*I*^2^ > 96%).

#### Total ‘High-Speed’ Distance

**Fig. 5:**
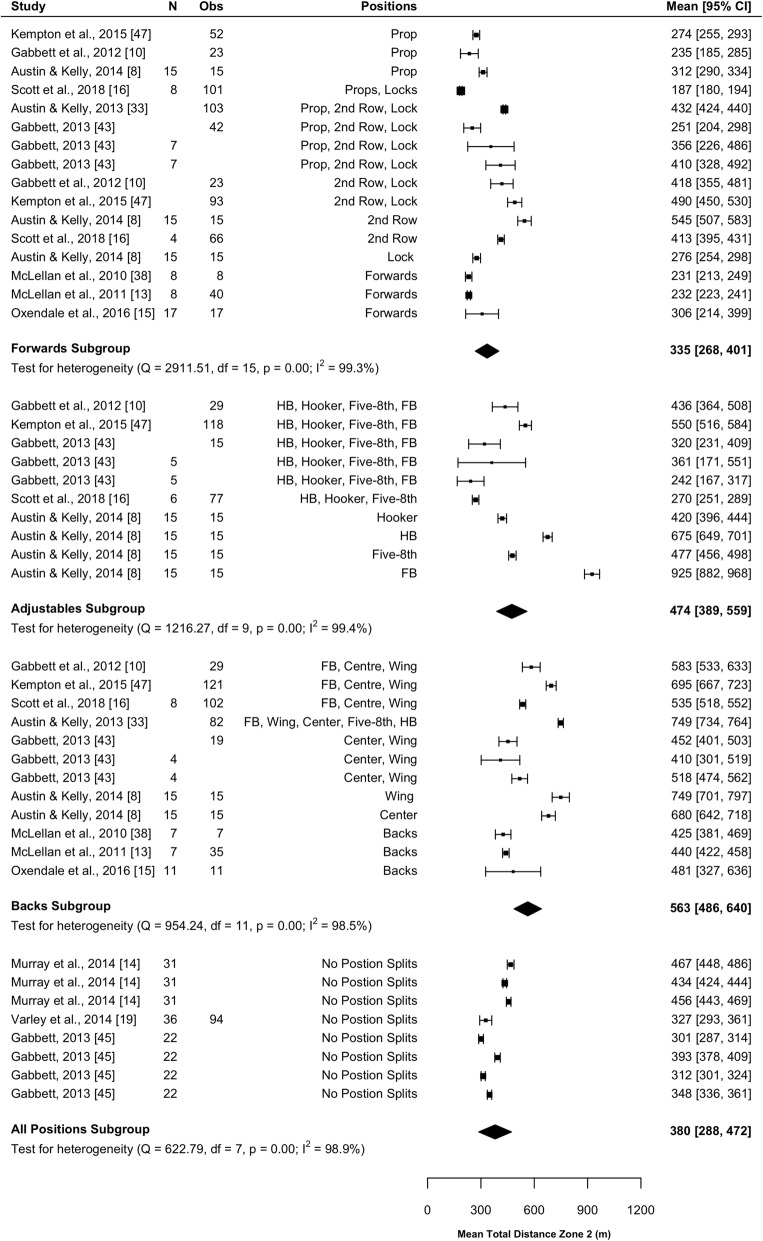
Meta-analysis of total high-speed distance. A forest plot (mean and 95% confidence intervals) was used to present the results of the meta-analysis and combined pooled estimates (random effects model). 2nd row, second row; HB, halfback; five-8th, five-eighth; FB, fullback, Obs, observations; CI, confidence interval

Thirteen [8, 10, 11, 13–16, 19, 33, 38, 43, 45, 47] studies (43%) reported total distance covered > 18 km·h^−1^. Up to three speed zones were included within a high-speed category, from 18 to 36 km·h^−1^. Results of the meta-analysis are presented in Fig. 5. Forwards cover significantly less high-speed distance during a full match than adjustables (mean difference [95%CI] 139 [31–247] m; *P* = 0.012) and backs (mean difference [95%CI] 229 [127–330] m; *P* < 0.001). There was no significant difference in high-speed distance between adjustables and backs (*P* = 0.127). All subgroups were associated with high heterogeneity (*I*^2^ > 98%).

#### Total Relative Distance

**Fig. 6:**
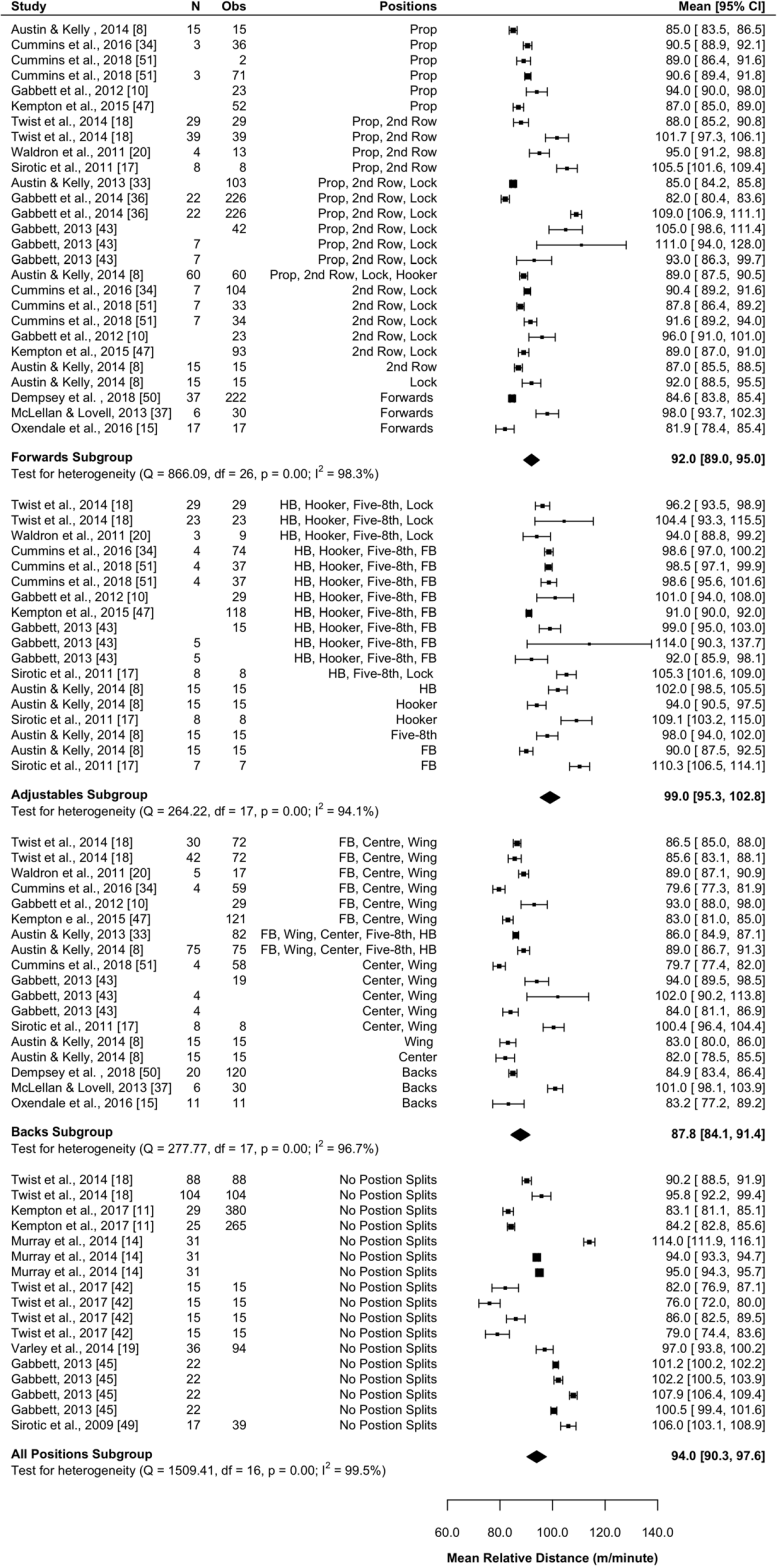
Meta-analysis of total relative distance. A forest plot (mean and 95% confidence intervals) was used to present the results of the meta-analysis and combined pooled estimates (random effects model). 2nd row, second row; HB, halfback; five-8th, five-eighth; FB, fullback, Obs, observations; CI, confidence interval

Nineteen studies (63%) [8, 10, 11, 15, 17–20, 33, 34, 36, 37, 42, 43, 45, 47, 49–51] reported total relative distance (m·min^−1^). Results of the meta-analysis are presented in Fig. 6. Forwards on average covered significantly less relative distance than adjustables (mean difference [95%CI] 7.1 [2.3–11.8] m·min^−1^; *P* = 0.004) and tended to cover more relative distance than backs (mean difference [95%CI] 4.2 [− 0.5–8.9] m·min^−1^; *P* = 0.079). The adjustables covered significantly (*P* < 0.001) greater relative distance than the backs (mean difference [95%CI] 11.3 [6.0–16.5] m·min^−1^). All subgroups were associated with high heterogeneity (*I*^2^ > 94%).

#### Total Relative ‘Slow-Speed’ Distance

Eight studies (27%) reported total relative slow-speed (0–18 km·h^−1^) distance [11, 14, 15, 19, 36, 42, 43, 45]. The meta-analysis results are presented in Additional file 1: Figure S1 and indicate that the backs subgroup cover the least relative slow-speed distance (mean [95%CI]: 84.6 [74.2–95.0] m·min^−1^; *I*^2^ = 96.8%); however, this is not significantly different to the distance covered by adjustables (mean [95%CI] 94.3 [81.1–107.5] m·min^−1^; *P* = 0.258; *I*^2^ = 1.8%) or the forwards (mean [95%CI] 91.8 [83.2–100.4] m·min^−1^; *P* = 0.296; *I*^2^ = 99.4%). There were also no significant differences between forwards and adjustables (*P* = 0.755). The adjustables subgroup was associated with low heterogeneity (*I*^2^ = 1.8%); all other subgroups were associated with high heterogeneity (*I*^2^ > 96%).

#### Total Relative ‘High-Speed’ Distance

Seven studies (23%) reported total relative high speed (> 18 km·h^−1^) [11, 14, 15, 19, 36, 42, 43, 45]. Results of the meta-analysis are presented in Additional file 1: Figure S2 and indicate that the backs cover the greatest relative distance at high speed (mean [95%CI] 5.5 [2.6–8.3] m·min^−1^; *I*^2^ = 85.6%); however, this was not significantly different from the forwards (mean [95%CI]: 4.8 [2.5–7.1] m·min^−1^; *P* = 0.889; *I*^2^ = 80.9%), or adjustables (mean [95%CI] 4.5 [1.2–7.8] m·min^−1^; *P* = 0.675; *I*^2^ = 86.3%). No significant differences were observed between the forwards and adjustables (*P* = 0.889). All subgroups were associated with high heterogeneity (*I*^2^ > 80%).

#### Total Repeat High-Intensity Efforts

Eight studies (27%) reported the total number of RHIE [10, 14, 15, 19, 35, 40, 43, 45]. Results of the meta-analysis are presented in Additional file 1: Figure S3 and indicate that forwards (mean [95%CI] 10.6 [8.7–12.5]; *I*^2^ = 96.1%) complete the greatest number of RHIE over a full match; however, this was not significant when compared to the adjustables (mean [95%CI] 10.4 [7.9–12.8]; *P* = 0.865; *I*^2^ = 95.2%), and backs subgroups (mean [95%CI] 9.6 [7.4–11.7]; *P* = 0.475; *I*^2^ = 98.4%). The difference between adjustables and backs was also not significant (*P* = 0.636). All subgroups were associated with high heterogeneity (*I*^2^ > 95%).

#### Total Relative Repeat High-Intensity Efforts

**Fig. 7:**
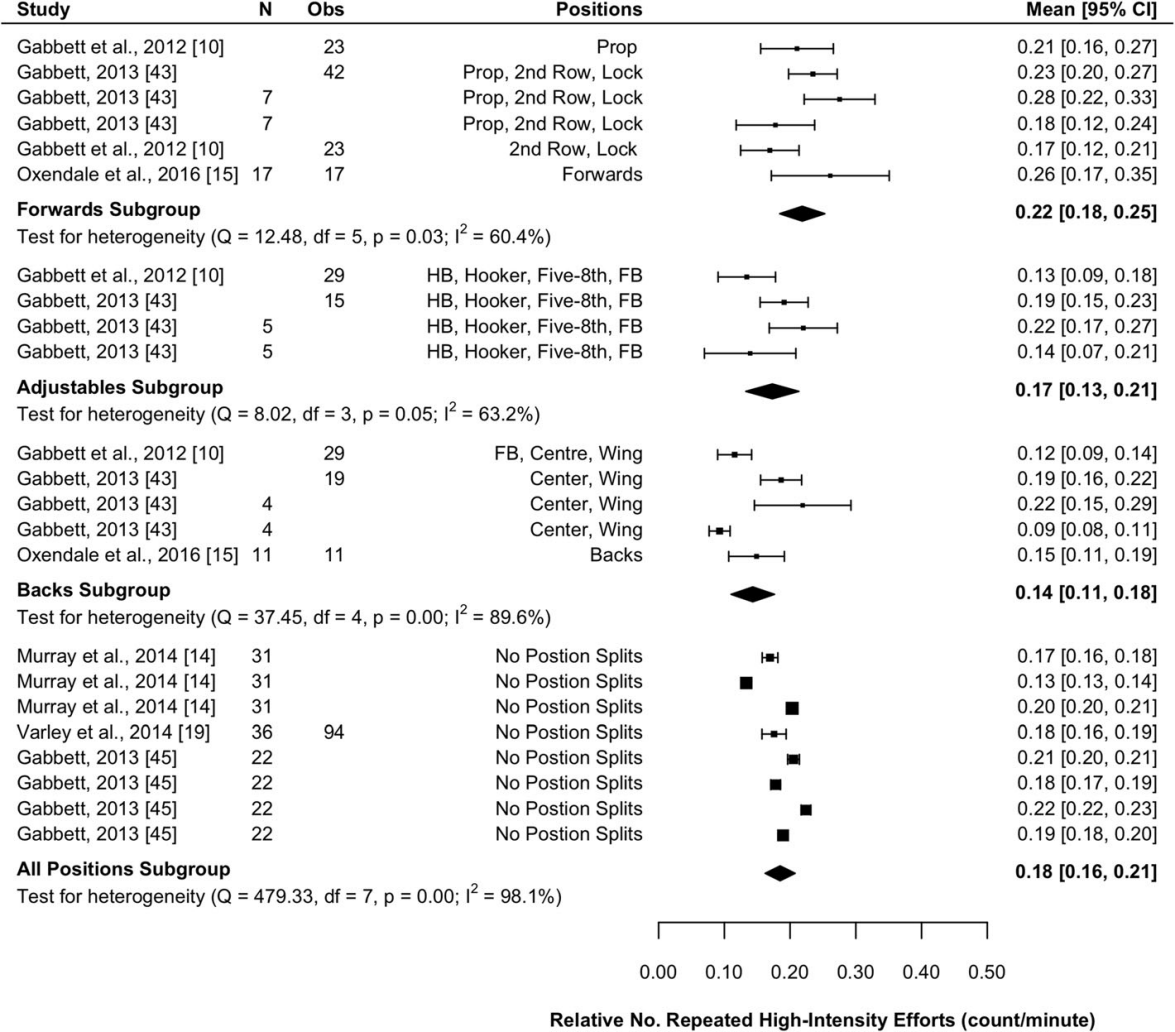
Meta-analysis of total relative repeated high-intensity efforts (RHIE). A forest plot (mean and 95% confidence intervals) was used to present the results of the meta-analysis and combined pooled estimates (random effects model). 2nd row, second row; HB, halfback; five-8th, five-eighth; FB, fullback, Obs, observations; CI, confidence interval

Six studies (20%) reported the total number of RHIE and playing time to allow calculation of total relative RHIE [10, 14, 15, 19, 43, 45]. Results of the meta-analysis are presented in Fig. 7 and indicate that forwards complete significantly more total relative RHIE than backs (mean difference [95%CI] 0.08 [0.03–0.12] n·min^−1^; *P* = 0.002) and tend to complete more than adjustables (mean difference [95%CI] 0.05 [− 0.01–0.10] n·min^−1^; *P* = 0.092). There was no significant difference between backs and adjustables (*P* = 0.263). All subgroups were associated with high heterogeneity (*I*^2^ > 60%).

#### Total Efforts Per Repeat High-Intensity Efforts

Two studies (7%) reported the number of total efforts per RHIE [10, 43]. Results of the meta-analysis are presented in Additional file 1: Figure S4 and indicate that forwards (mean [95%CI] 5.6 [4.0–7.1]; *I*^2^ = 97.8%) perform the greatest number of efforts per RHIE; however, this was not significant when compared to the adjustables (mean [95%CI] 5.2 [3.4–6.9]; *I*^2^ = 98.9%) (*P* = 0.745) and backs subgroups (mean [95%CI] 5.4 [3.7–7.1]; *I*^2^ = 98.6%) (*P* = 0.889). No significant differences were observed between the adjustables and backs (*P* = 0.860). All subgroups were associated with high heterogeneity (*I*^2^ = 95%).

#### Total Accelerations

Three studies (10%) reported the total number of accelerations [11, 45, 47]. Results of the meta-analysis are presented in Additional file 1: Figure S5. One outlier study [15] was removed from both the forwards (mean [95%CI] 4.7 [3.3–6.1]) and backs (mean [95%CI] 9.1 [5.3–12.9]) subgroups. The adjustables subgroup showed the highest number of total accelerations (mean [95%CI] 78.3 [29.2–127.4]; *I*^2^ = 0.0%); however, this was not significant compared to the forwards subgroup (mean [95%CI] 58.7 [24.0–93.4]; *I*^2^ = 96.5%) (*P* = 0.524) or backs subgroup (mean [95%CI] 65.8 [16.7–114.9]; *I*^2^ = 0.0%) (*P* = 0.724). No significant differences were observed between the forwards and backs (*P* = 0.818). The adjustables and backs subgroups were associated with low heterogeneity (*I*^2^ = 0.0%), whereas the forwards and all positions subgroup were associated with high heterogeneity (*I*^2^ > 96%).

#### Total Decelerations

Three studies (10%) reported total decelerations [11, 15, 47]. Results of the meta-analysis (mean [95%CI]) are presented in Additional file 1: Figure S6. One outlier study [15] was removed from both the forwards (mean [95%CI] 8.4 [6.2–10.6]) and backs subgroups (mean [95%CI] 9.6 [6.2–13.0]). The adjustables subgroup displayed the most decelerations (mean [95%CI] 93.3 [68.8–117.8]; *I*^2^ = 0.0%); however, this was not significantly more than the forwards subgroup (mean [95%CI] 69.2 [51.9–86.5]; *I*^2^ = 98.1%) (*P* = 0.115), or than the backs subgroup (mean [95%CI] 82.7 [58.3–107.1]; *I*^2^ = 0.0%) (*P* = 0.548). The backs showed a greater number of decelerations than the forwards; however, this difference was not significant (*P* = 0.377). The adjustables and backs subgroups were associated with low heterogeneity (*I*^2^ = 0.0%), whereas the forwards and all positions subgroups were associated with high heterogeneity (*I*^2^ > 94%).

#### Total Collisions

**Fig. 8:**
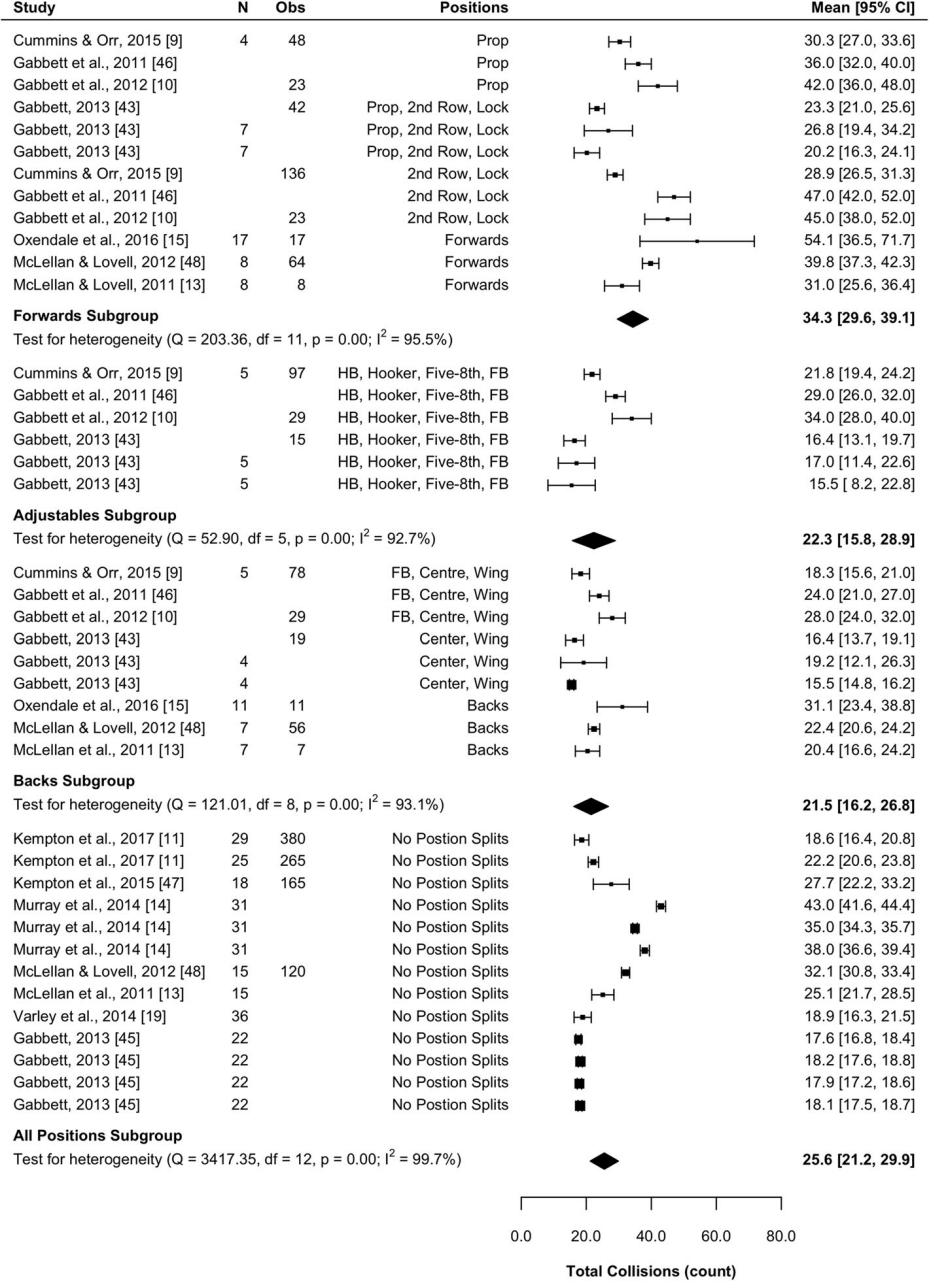
Meta-analysis of total collisions. A forest plot (mean and 95% confidence intervals) was used to present the results of the meta-analysis and combined pooled estimates (random effects model). 2nd row, second row; HB, halfback; five-8th, five-eighth; FB, fullback, Obs, observations; CI, confidence interval

Ten studies (33%) reported total collisions [9–11, 14, 19, 39, 43, 45, 46, 48]. Results of the meta-analysis are presented in Fig. 8 and indicate that forwards are involved in a significantly greater number of collisions than both the adjustables (mean difference [95%CI] 12.0 [3.9–20.1] collisions; *P* = 0.004) and backs (mean difference [95%CI] 12.8 [5.7–20.0] collisions; *P <* 0.001) subgroups. There was no significant difference between backs and adjustables (*P* = 0.847). All subgroups were associated with high heterogeneity (*I*^2^ > 92%).

#### Total ‘Low-Impact’ Collisions

Five studies (17%) reported the number of ‘low impact’ collisions [9, 10, 19, 43, 45]. Up to five collision magnitude zones were presented from 0 to 8 g. Results of the meta-analysis are presented in Additional file 1: Figure S7 and indicate that forwards (mean [95%CI] 14.1 [9.0–19.2] collisions; *I*^2^ = 99.4%) are involved in the most low impact collisions; however, this was not significantly more than the adjustables (mean [95%CI] 10.0 [3.8–16.1] collisions; *I*^2^ = 97.7%) (*P* = 0.311) or backs subgroups (mean [95%CI]: 6.7 [0.6–12.9] collisions; *I*^2^ = 96.6%) (*P* = 0.072). The adjustables are shown to be involved in more low impact collisions than the backs; however, this difference was not significant (*P* = 0.468). All position subgroups were associated with high heterogeneity (*I*^2^ > 96%), except for the all positions subgroup which was associated with low heterogeneity (*I*^2^ = 0.0%).

#### Total ‘High-Impact’ Collisions

Five studies (17%) reported the number of high impact collisions [9, 10, 19, 43, 45]. Up to two collision magnitude zones were presented from 8 to 15 g. Results of the meta-analysis are presented in Additional file 1: Figure S8 and indicate that the forwards (mean [95%CI] 16.0 [13.6–18.4] collisions; *I*^2^ = 92.9%) tended to be involved in the most high impact collisions, compared to the adjustables (mean [95%CI] 12.4 [9.7–15.1] collisions; *P* = 0.053; *I*^2^ = 84.6%) and backs (mean [95%CI] 13.0 [10.5–15.5] collisions; *P* = 0.091; *I*^2^ = 14.5). There was no significant difference between backs and adjustables (*P* = 0.763). The backs subgroup was associated with low heterogeneity (*I*^2^ = 14.5%). All other subgroups were associated with high heterogeneity (*I*^2^ > 63%).

#### Total Relative Collisions

**Fig. 9:**
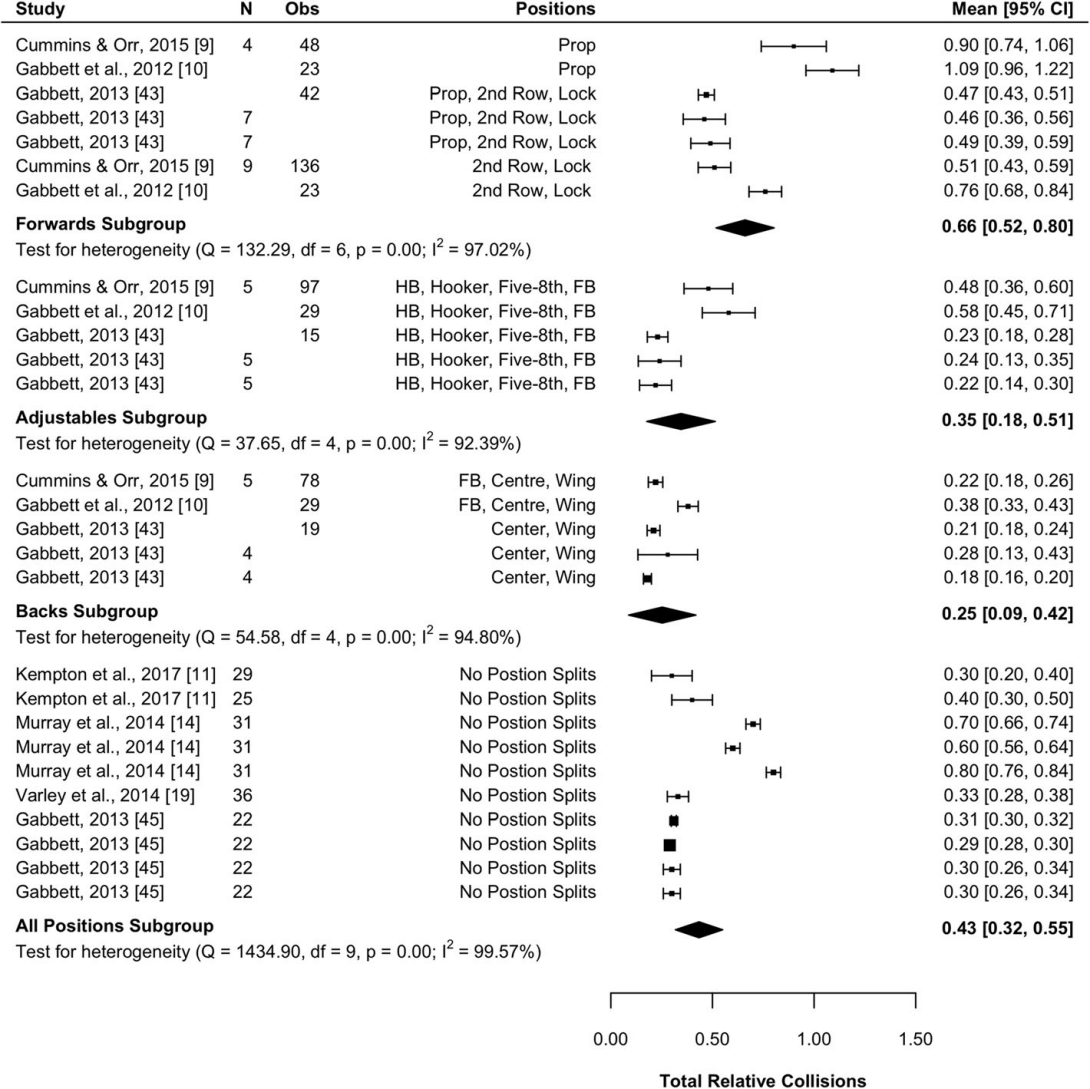
Meta-analysis of total relative collisions. A forest plot (mean and 95% confidence intervals) was used to present the results of the meta-analysis and combined pooled estimates (random effects model). 2nd row, second row; HB, halfback; five-8th, five-eighth; FB, fullback, Obs, observations; CI, confidence interval

Seven studies (23%) reported total relative collisions [9–11, 14, 19, 43, 45]. Results of the meta-analysis are presented in Fig. 9 and indicate that forwards are involved in significantly more relative collisions than adjustables (mean difference [95%CI] 0.32 [0.09–0.54] n·min^−1^; *P* = 0.005) and backs (mean difference [95%CI] 0.41 [0.19–0.63] n·min^−1^; *P* = 0.003). No significant differences were observed between the adjustables or backs for relative collisions (*P* = 0.444). All subgroups were associated with high heterogeneity (*I*^2^ > 92%).

## Discussion

The purpose of this review was to characterise the demands of professional rugby league, across three positional groups (forwards, adjustables and backs). After a systematic search, 30 studies were included in the meta-analysis, and 16 variables could be synthesised across the studies. Significant differences were observed between positional groups in 8 of the 16 variables. Meta-analysis demonstrated that, compared to the adjustables and backs, forwards spend the least amount of time on the field and cover the least total, slow-speed and high-speed distance, but are involved in the most relative RHIEs, and total and relative collisions. Notably, when distance was expressed relative to playing time, the forwards were not different to adjustables and backs in slow-speed and high-speed relative distance. The adjustables and backs subgroups were similar in most variables, except playing time (shorter for adjustables), slow-speed distance (greater for adjustables) and total relative distance (greater for adjustables). There were no significant differences between any positional group for total efforts per RHIE, total accelerations and decelerations.

To the authors’ knowledge, this is the second meta-analysis investigating the physical characteristics of rugby league match demands but the first to focus on only professional rugby league [28]. Characterising professional rugby league is particularly important because a thorough understanding of professional match demands by sport scientists and coaches can facilitate improved planning and training practices. Non-professional teams may also use these data as a means of comparing themselves to the highest level in order to set benchmarks for their performance. Despite limiting the review to professional rugby league, there were four more studies included than the previous review that included studies with non-professional athletes. Synthesising a large number of studies is important for determining the physical characteristics of professional rugby league matches as previous studies in this area have used convenience samples from only one or two clubs. This makes it difficult to generalise the findings from these individual studies given the differing match tactics and physical conditioning priorities between clubs. Findings of this review are more generalisable than the individual studies as they are based on estimates from multiple clubs.

The forwards subgroup demonstrated the most unique match characteristics amongst the investigated subgroups. Forwards had the shortest playing time and match distance, were involved in the most collisions, and completed the most RHIE per minute. Collisions and RHIE are a highly taxing aspect of rugby league match-play [52], and it is likely that the associated fatigue experienced by forwards can be attributed to involvement in these match demands. Indeed, previous research in semi-professional [29, 30] and junior elite [31] rugby league has demonstrated that RHIE and collisions reduce running intensity and skill efficiency, and cause neuromuscular fatigue and reduced power. The greater involvement of forwards in collisions and RHIE than the other position groups is consistent with the higher number of interchanges completed by the forwards [12, 53]. A rugby league team is permitted between 8 and 10 player interchanges and the positional demands of professional rugby league dictate that these interchanges are used to allow forwards time to recover from the high rate of collisions and RHIE. Our findings suggest that forwards typically complete 8–13 RHIE and experience 30–40 collisions per game, which may provide target values that can be used to guide interchanges and identify underperforming players. These RHIE and collisions typically occur at a rate of 2–3 RHIE and 5–8 collisions every 10 min of playing time, although peak 10-min match demands can be higher than these rates. This information should be considered when designing conditioning drills intended to simulate the demands of rugby league matches for forwards. Collisions are also major causation of injuries such as concussion [54, 55], and understanding the collision demands of professional rugby league may lead to improved prevention and management of such injuries.

The forwards covered less absolute slow-speed, high-speed and total distance compared to the adjustables and backs; however, this pattern was not maintained when the distance was expressed relative to playing time. Slow-speed and high-speed relative distance were not different across positions, while total relative distance was less for forwards compared to adjustables and tended to be greater for forwards compared to backs. These findings highlight that both absolute and relative distance variables should be used to compare between playing positions to provide a clearer understanding of the different match demands. Training characteristics and conditioning drills can then be tailored to the specific playing position match demands. The absolute and relative distance findings from this review indicate that forwards should be prescribed less absolute slow-speed and high-speed distance than other position groups, but should complete prescribed distances at the same rate per minute. Adjustables and backs need to be adequately prepared to run a greater total, slow-speed and high-speed distance than forwards.

In all but 3 of the 16 synthesised variables in this review, the adjustables and backs showed no significant differences. The only differences observed were less playing time and absolute slow-speed distance but more total relative distance for adjustables compared to backs. As a result, the physical demands for adjustables and backs are very similar within a match and their general physical preparation can also be similar. However, despite the equivalent results for the movement variables considered in this review, there are known differences in the tactical demands of adjustables and backs. Backs can be characterised by longer and relatively more steady-state bouts of activity, where they have larger areas of open space to cover with the ball at high speeds [13, 33]. In contrast, adjustables can be characterised by brief, more intense periods of activity and a greater number of contacts with the ball as play-makers [20]. Considering these characteristics, backs may be expected to cover more high-speed distance than the adjustables. However, it may be that the large distances covered at high speed by backs is equalled by a greater number of short sprints at high speed by adjustables, as evidenced by more (non-significant) accelerations, and RHIE for adjustables than backs shown in this review. It is more common to tactically substitute an adjustable at select stages of a match, than to substitute a back [53], which accounts for the reduction in playing time.

The present review found only four studies to have reported on total accelerations and/or total decelerations [11, 15, 45, 47] and pooled results from these studies demonstrated no differences between playing positions. Four was a surprisingly small number of studies, considering the repeated acceleration and deceleration nature of the sport. The low number of studies may be attributed to concerns about the validity and reliability of accelerations and decelerations derived from some GPS devices [56]. Notably, recent research indicates that assessing the average accelerations of each player, as opposed to total accelerations, may be a more beneficial measure of match intensity and facilitate quantification of the energetic costs of running and acceleration efforts [57]. Further research involving average accelerations and energetics running costs of rugby league athletes will facilitate future meta-analyses on this topic and assist coaches with training prescription and monitoring.

There were several limitations in the current literature characterising the movement in professional rugby league match-play that were evident in this review. First, it is evident that there is no consensus on classifying the parameters of different speed zones because both the terminology and speed parameters vary greatly across studies. This made it difficult to directly compare distances covered in speed zones across studies, and several studies had to be excluded from the meta-analysis due to speed zones falling outside of the low and high-speed parameters adopted in this review. Second, not all GPS units used in the included studies have been validated for detecting collisions. Third, there is a strong bias towards studies reporting on players from the Australian National Rugby League (27/30 studies) compared to players from other professional rugby league competitions. As such, findings of this review should be generalised with caution to non-Australian rugby league players because of potential differences in styles of play and rules across different competitions. For example, when scores are equal at the end of regular time, the Australian National Rugby League has enforced a “golden-point” (first score wins) extra time rule since 2003, whereas the English Super League will adopt this rule for the first time in the 2019 season. This rule affects the playing time in a match for each player and, in turn, every playing variable. Last, some studies were not explicit in stating participant numbers, or the number of observation files recorded for each participant or position group. Future research should ensure that both participant numbers and number of observation files are reported within the study methods.

There are also several limitations to this review that should be considered when interpreting the present results. First, the majority of the outcomes from this meta-analysis were associated with high statistical heterogeneity. This indicates that there are large variations in study outcomes across the different studies reported in the literature. It is possible that this is caused by all studies observing players from only one club and each club has unique tactical and physical conditioning practices. The high statistical heterogeneity could also be caused by the variety of GPS manufacturers and models used across studies to record the movement data presented in this review. Second, this review focused on overall match demands not peak demands or specific periods of a match (e.g. halves or quarters). Focusing on overall match demands facilitated the inclusion of more studies but information about peak demands or specific periods is important for game strategy and player conditioning. Future reviews on this topic should seek to investigate these aspects of rugby league match-play. Additionally, future research should consider even more movement variables than are presented in this review, such as metabolic power, which might provide further insight.

## Conclusions

In conclusion, this review characterised the demands of professional rugby league, across three positional groups. The results of this review will assist both professional strength and conditioning and tactical coaches, alike, to better understand the demands of the game, specific to each position. The results of this review demonstrate significant differences between position groups in half of the synthesised, match-specific movement variables. These results highlight unique match demands for each playing position that should be considered when planning training programmes and developing game strategies for professional rugby league. Future research characterising the demands of rugby league match-play should look to include both absolute and relative (to playing time) measures, to provide a clear understanding of the different match-demands without bias to any playing position.

## Additional Files

Additional file 1: **Table S1.** Results of study methodological quality assessment. **Figure S1.** Total relative slow-speed distance forest plot. **Figure S2.** Total relative high-speed distance forest plot. **Figure S3.** Total repeat high-intensity efforts forest plot. **Figure S4.** Total efforts per repeat high-intensity effort forest plot. **Figure S5.** Total accelerations forest plot. **Figure S6.** Total decelerations forest plot. **Figure S7.** Total ‘low impact’ collisions forest plot. **Figure S8.** Total ‘high impact’ collisions forest plot (DOCX 2831 kb)

Additional file 2: **Appendix 1.** Analysed datasets. (XLSX 55 kb)

## Data Availability

The datasets generated during and/or analysed during the current systematic review are available in Additional file 2: Appendix 1.

## Abbreviations

AD: Adjustables
B: Backs
CI: Confidence interval
F: Forwards
GPS: Global positioning system
LST: Less-successful team
NR: Not reported
NRL: National Rugby League
RHIE: Repeat high-intensity effort
ST: Successful team
