# Supplementary material for: The Demands of Professional Rugby League Match-Play: a Meta-analysis
Source: Sports Med Open. 2019 Jun 11;5:24. doi: 10.1186/s40798-019-0197-9 (PMC6560119; doi:10.1186/s40798-019-0197-9)
Supplement: Supplementary file 1 — Table S1. Results of study methodological quality assessment. Figure S1. Total relative slow-speed distance forest plot. Figure S2. Total relative high-speed distance forest plot. Figure S3. Total repeat high-intensity efforts forest plot. Figure S4. Total efforts per repeat high-intensity effort forest plot. Figure S5. Total accelerations forest plot. Figure S6. Total decelerations forest plot. Figure S7. Total ‘low impact’ collisions forest plot. Figure S8. Total ‘high impact’ collisions forest plot (DOCX 2831 kb) [file 40798_2019_197_MOESM1_ESM.docx]

**THE DEMANDS OF PROFESSIONAL RUGBY LEAGUE MATCH-PLAY: A META-ANALYSIS**

**Electronic Supplementary Material Table and Figures**

Daniel J. Glassbrook^1^, Tim L.A. Doyle^1^, Jacqueline A. Alderson^2^, and Joel T. Fuller^1^

^1^Faculty of Medicine and Health Sciences, Macquarie University, NSW, Australia.

^2^The University of Western Australia, Perth, Australia.

Daniel J. Glassbrook ORCID: 0000-0002-3317-8791

Tim L.A. Doyle ORCID: 0000-0002-1227-6835

Jacqueline A. Alderson ORCID: 0000-0002-8866-0913

Joel T. Fuller ORCID: 0000-0002-0997-4878

**Corresponding Author:** Dr Joel Fuller

Department of Health Professions

75 Talavera Road, Macquarie Park 2113

Macquarie University

Phone: + 61 2 9850 4040

Email: joel.fuller@mq.edu.au

**Table S1. Results of Study Methodological Quality Assessment**

|  | **Criteria** | | | | | | | | | | | | |
| --- | --- | --- | --- | --- | --- | --- | --- | --- | --- | --- | --- | --- | --- |
| **Study** | **1** | **2** | **3** | **4** | **5** | **6** | **7** | **8** | **9** | **10** | **11** | **12** | **Score/12** |
| Austin et al. (2011) [40] | Y | Y | Y | Y | Y | Y | N | N | N | Y | Y | Y | 9 |
| Austin & Kelly (2013) [33] | Y | Y | Y | Y | Y | Y | N | N | N | Y | Y | Y | 9 |
| Austin & Kelly (2014) [8] | Y | Y | Y | Y | Y | Y | N | N | N | Y | Y | Y | 9 |
| Cummins et al. (2016) [34] | Y | Y | Y | Y | Y | Y | Y | N | N | Y | Y | Y | 10 |
| Cummins et al. (2017) [51] | Y | Y | Y | Y | Y | Y | Y | N | N | Y | Y | Y | 10 |
| Cummins & Orr (2015) [9] | Y | Y | Y | Y | Y | Y | Y | N | N | Y | Y | Y | 10 |
| Dempsey et al. (2018) [50] | Y | Y | Y | Y | Y | Y | Y | N | N | Y | Y | Y | 10 |
| Gabbett (2012) [35] | Y | Y | Y | Y | Y | Y | N/A | N | N | Y | N | Y | 8 |
| Gabbett (2013) [45] | Y | Y | N | Y | Y | Y | N/A | U | U | Y | Y | Y | 8 |
| Gabbett (2013) [43] | Y | Y | N | Y | Y | Y | N/A | U | U | Y | Y | Y | 8 |
| Gabbett et al. (2011) [46] | Y | Y | Y | Y | Y | Y | N/A | N | N | Y | Y | Y | 9 |
| Gabbett et al. (2012) [10] | Y | Y | Y | Y | Y | Y | N/A | N | N | Y | Y | Y | 9 |
| Gabbett et al. (2014) [36] | Y | Y | Y | Y | Y | Y | N/A | N | N | Y | Y | Y | 9 |
| Kempton et al. (2017) [11] | Y | Y | Y | Y | Y | Y | N/A | N | N | Y | Y | Y | 9 |
| Kempton et al. (2015) [47] | Y | Y | Y | Y | Y | Y | N/A | N | N | Y | Y | Y | 9 |
| King et al. (2009) [12] | Y | Y | N | Y | Y | Y | Y | U | U | Y | Y | Y | 9 |
| McLellan & Lovell (2012) [48] | Y | Y | Y | Y | Y | Y | Y | N | N | Y | Y | Y | 10 |
| McLellan & Lovell (2013) [37] | Y | Y | N | Y | Y | Y | N | U | U | Y | Y | Y | 8 |
| McLellan et al. (2010) [38] | Y | Y | Y | Y | Y | Y | N | N | N | Y | Y | Y | 9 |
| McLellan et al. (2011) [13] | Y | Y | Y | Y | Y | Y | N | N | N | Y | Y | Y | 9 |
| McLellan et al. (2011) [39] | Y | Y | Y | Y | Y | Y | N | N | N | Y | Y | Y | 9 |
| Murray et al. (2014) [14] | Y | Y | N | Y | Y | Y | N/A | N | N | Y | Y | Y | 8 |
| Oxendale et al. (2016) [15] | Y | Y | Y | Y | Y | Y | N | N | N | Y | Y | Y | 9 |
| Scott et al. (2018) [16] | Y | Y | Y | Y | Y | Y | N/A | N | N | Y | Y | Y | 9 |
| Sirotic et al. (2009) [49] | Y | Y | N | Y | Y | Y | Y | N | N | Y | Y | Y | 9 |
| Sirotic et al. (2011) [17] | Y | Y | Y | Y | Y | Y | Y | N | N | Y | Y | Y | 10 |
| Twist et al. (2017) [42] | Y | Y | Y | Y | Y | Y | N/A | N | N | Y | Y | Y | 9 |
| Twist et al. (2014) [18] | Y | Y | N | Y | Y | Y | Y | N | N | Y | Y | Y | 9 |
| Varley et al. (2014) [58] | Y | Y | N | Y | Y | Y | N/A | N | N | Y | Y | Y | 8 |
| Waldron et al. (2011) [20] | Y | Y | Y | Y | Y | Y | Y | N | N | Y | Y | Y | 10 |
| N/A, Not applicable as effect sizes were give; U, Unable to determine, Y, Yes; N, No. Criteria: 1, hypothesis/aim clearly stated; 2, main outcomes described in introduction or method; 3, subject characteristics described; 4, intervention described; 5, main findings described; 6, estimate of random variability for main outcome; 7, actual probability value reported; 8, potential recruits representative of entire population; 9, participants representative of entire population from which they were recruited; 10, data dredging made clear; 11, statistical tests appropriate; 12, outcome measures valid and reliable. | | | | | | | | | | | | | |

**Figure S1. Total Relative ‘Slow Speed’ Distance Forest Plot**

**
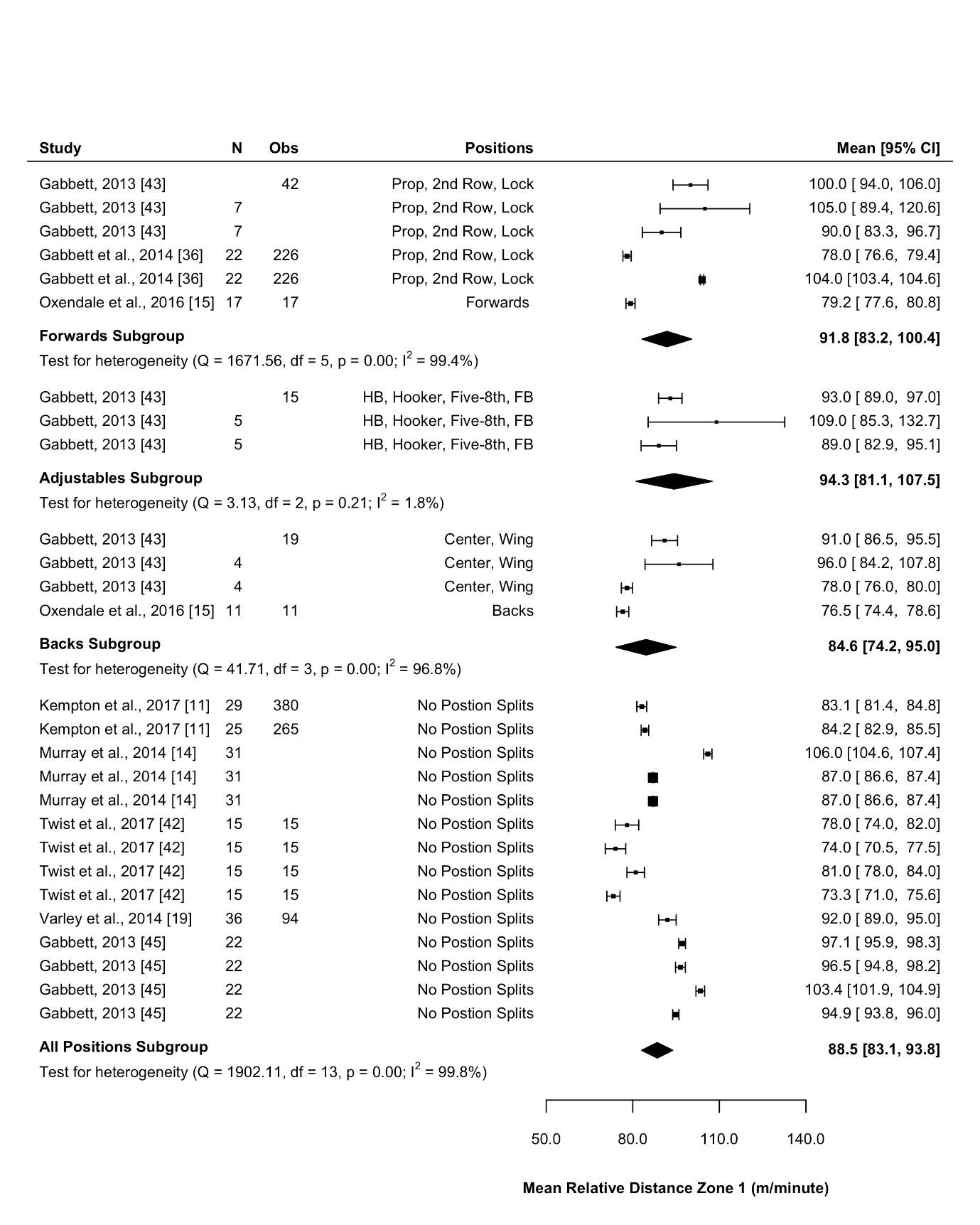
**

**Figure S2. Total Relative ‘High Speed’ Distance Forest Plot**


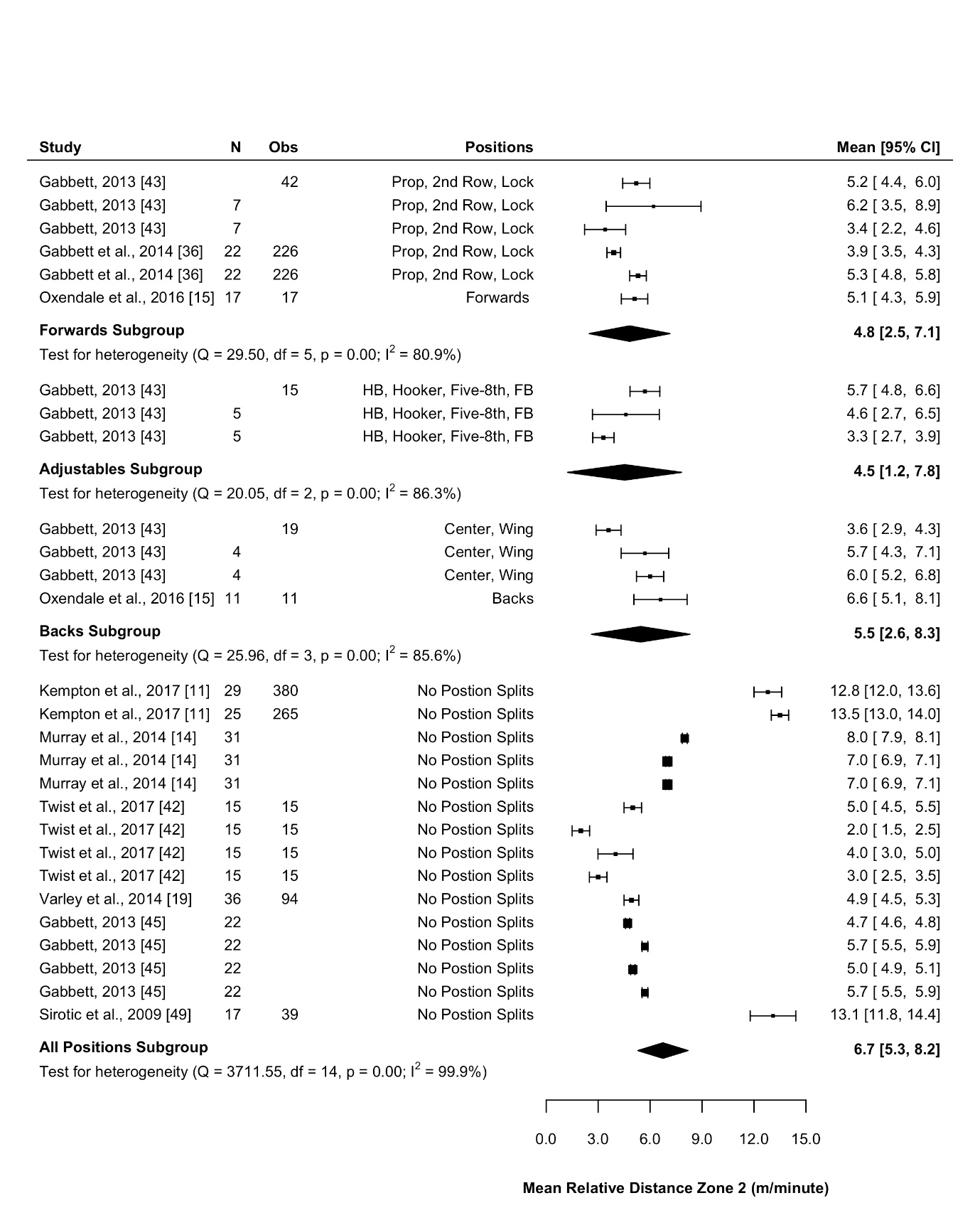


**Figure S3. Total Repeat High Intensity Efforts Forest Plot**

**
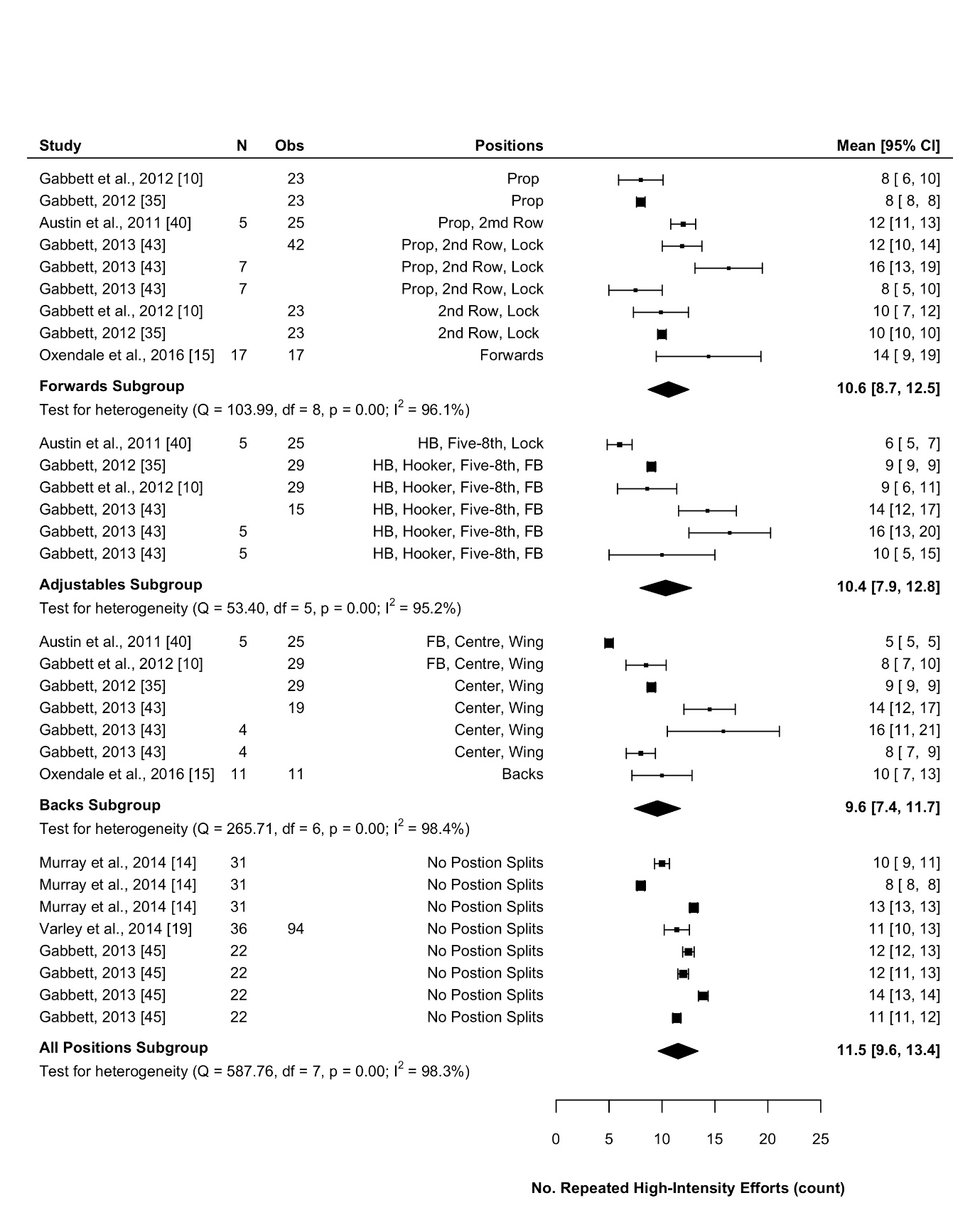
**

**Figure S4. Total Efforts Per Repeat High Intensity Effort Forest Plot**

**
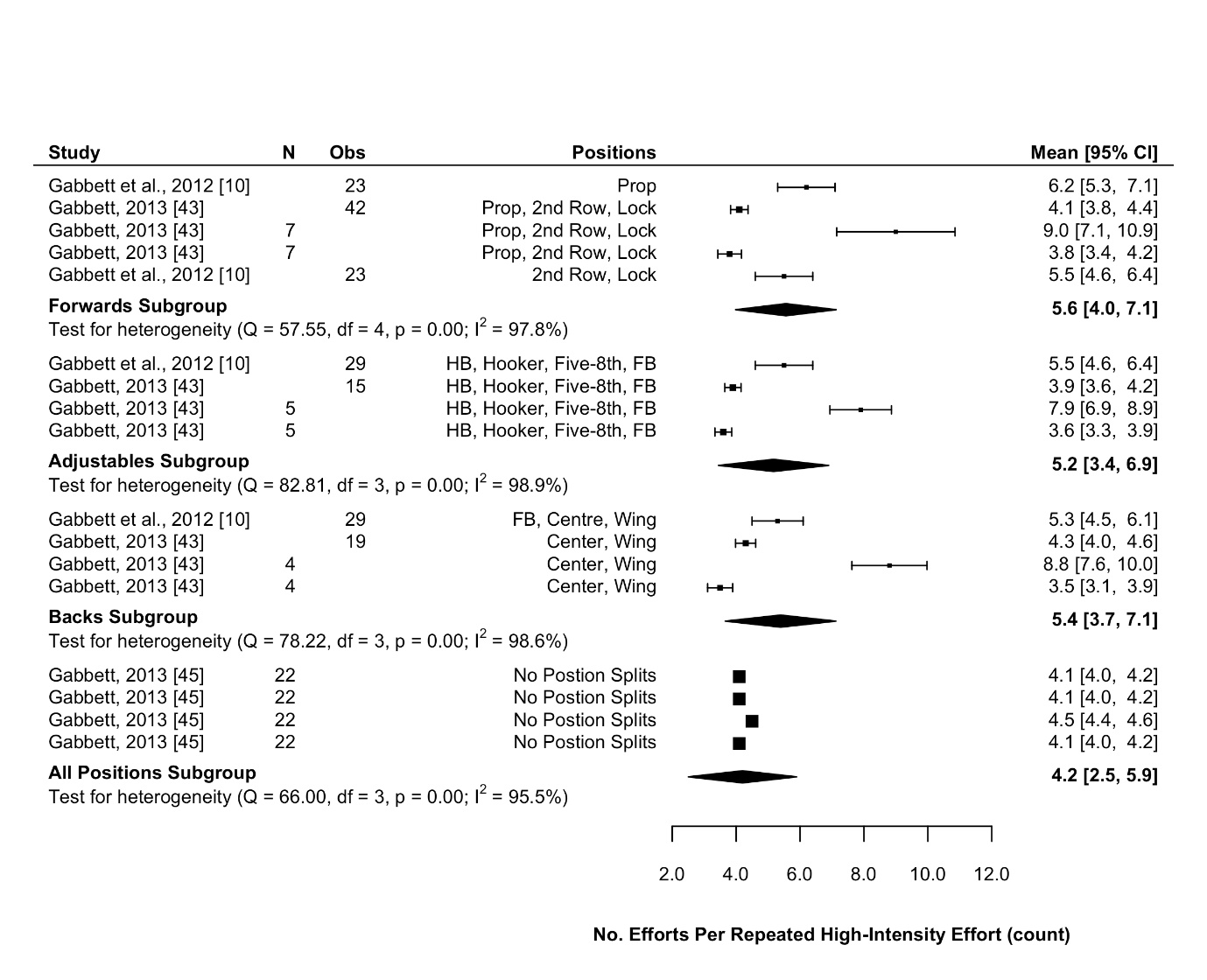
**

**Figure S5. Total Accelerations Forest Plot**


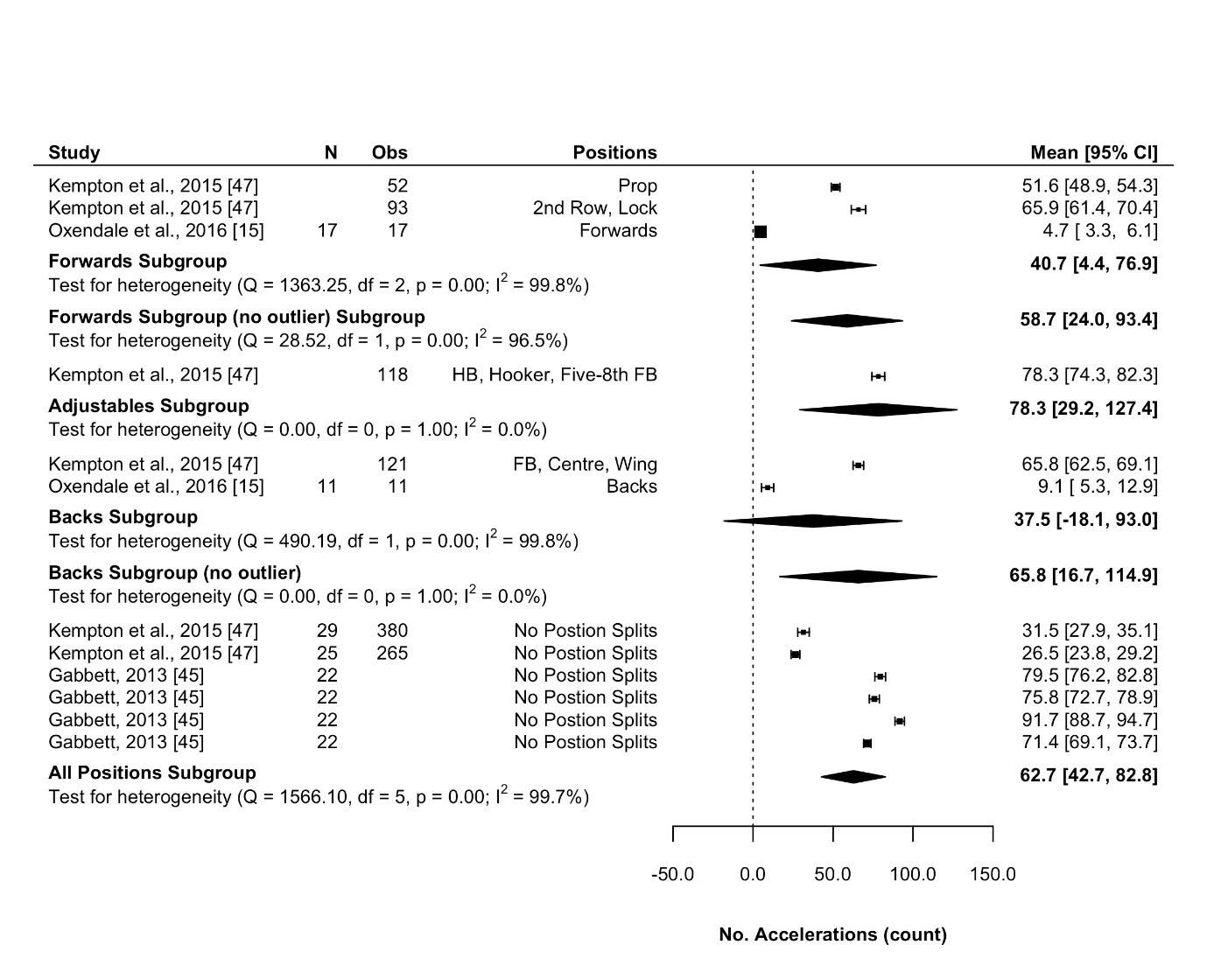


**Figure S6 Total Decelerations Forest Plot**

**
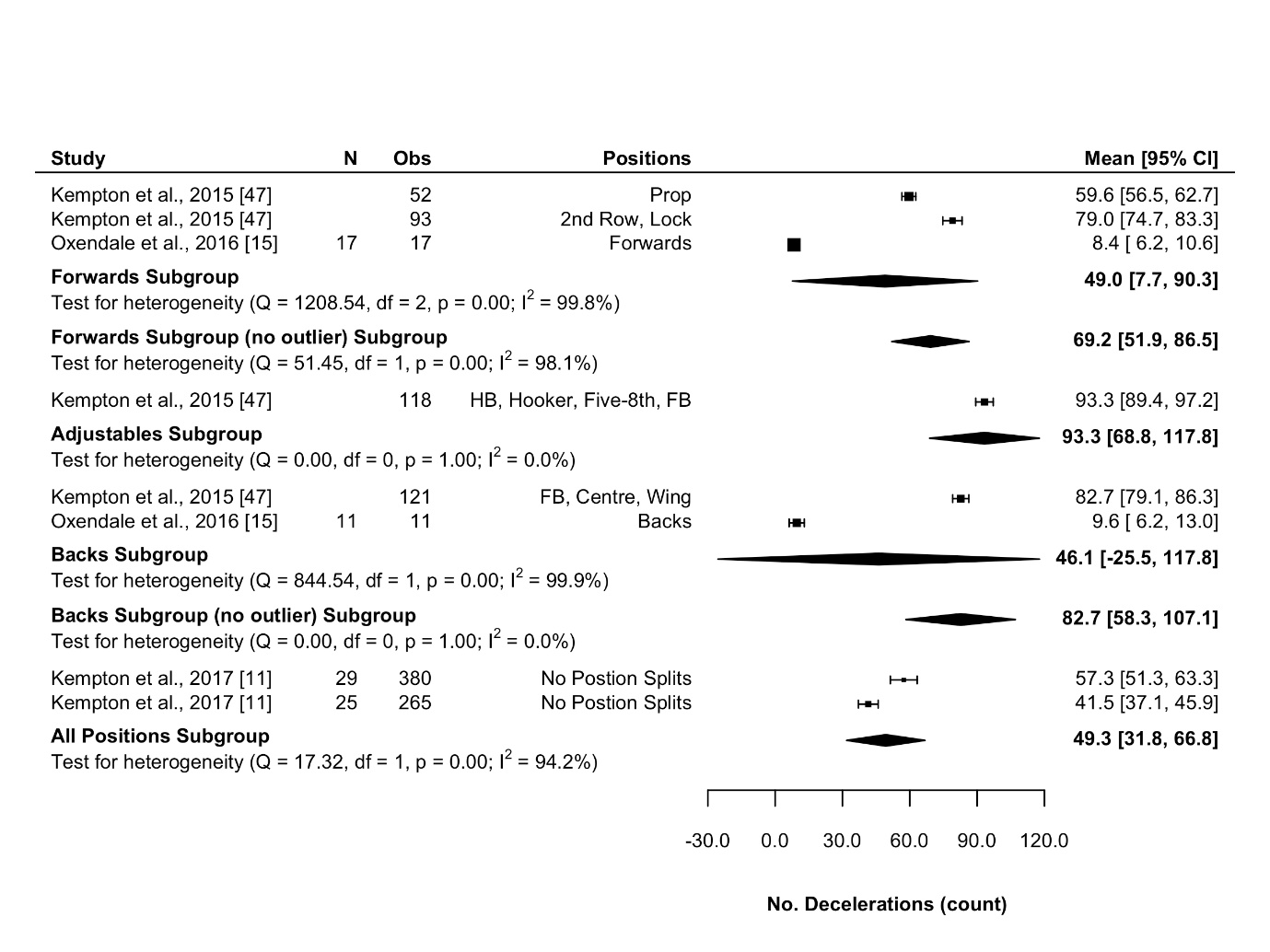
**

**Figure S7. Total ‘Low Impact’ Collisions Forest Plot**

**
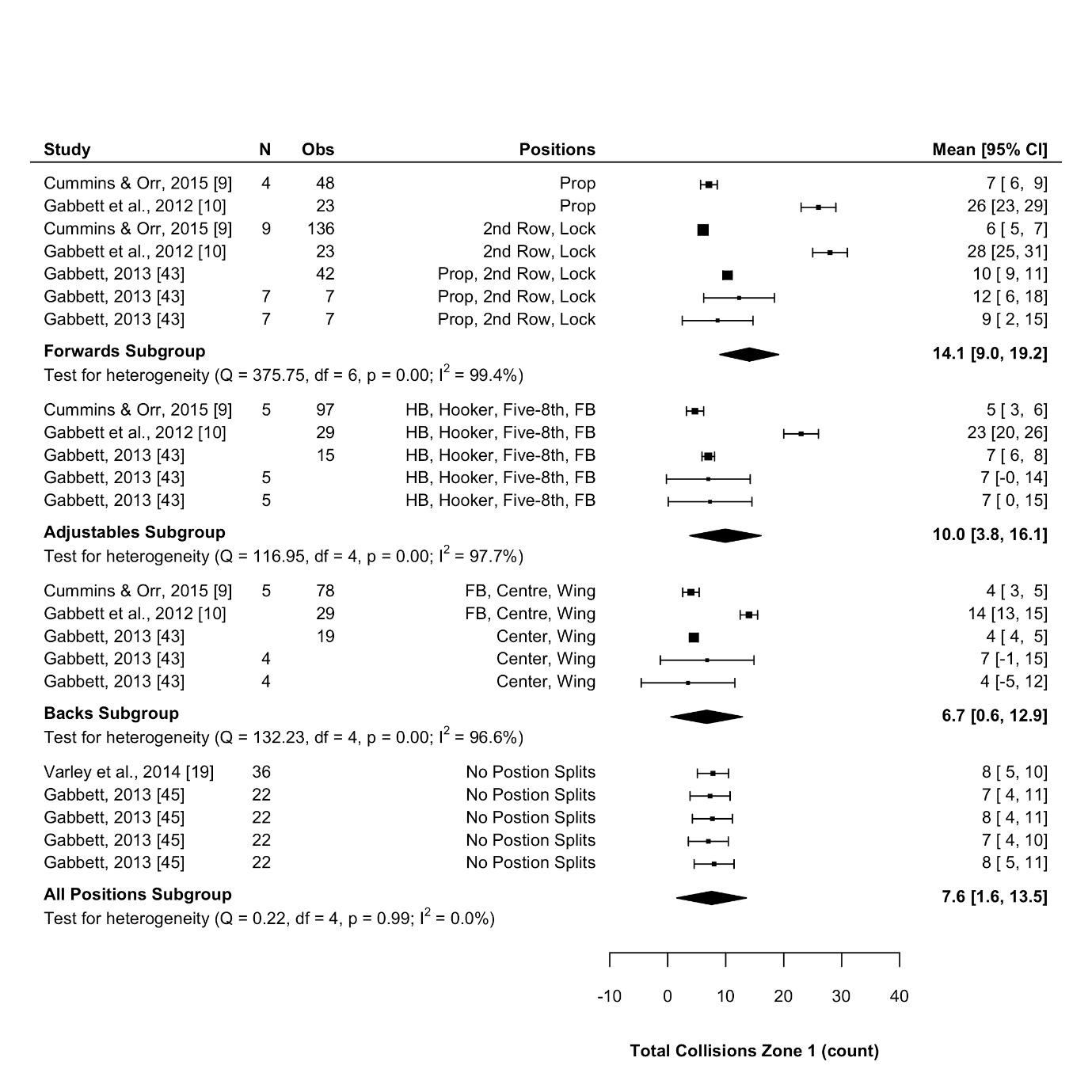
**

**Figure S8. Total ‘High Impact’ Collisions Forest Plot**

**
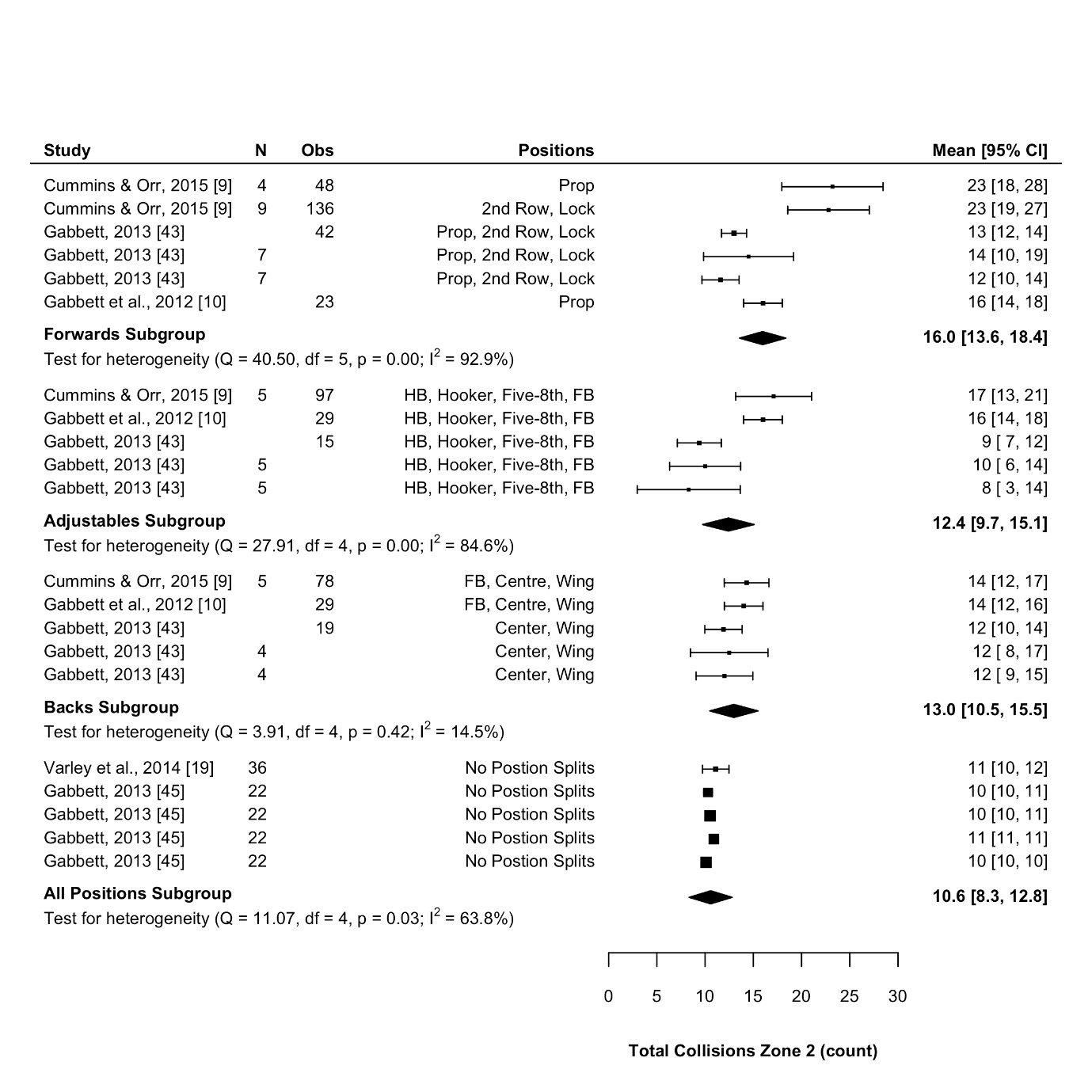
**
